# Copy number variation in the *MSRB3* gene enlarges porcine ear size through a mechanism involving miR-584-5p

**DOI:** 10.1186/s12711-018-0442-6

**Published:** 2018-12-27

**Authors:** Congying Chen, Chenlong Liu, Xinwei Xiong, Shaoming Fang, Hui Yang, Zhiyan Zhang, Jun Ren, Yuanmei Guo, Lusheng Huang

**Affiliations:** 1 State Key Laboratory of Pig Genetic Improvement and Production Technology, Jiangxi Agricultural University, Nanchang, 330045 China; 2 Institute of Animal Husbandry and Veterinary, Jiangxi Academy of Agricultural Sciences, Nanchang, 330200 China

## Abstract

**Background:**

The size and type of ears are important conformation characteristics that distinguish pig breeds. A significant quantitative trait locus (QTL) for ear size has been identified on SSC5 (SSC for *Sus scrofa* chromosome) but the underlying causative gene and mutation remain unknown. Thus, our aim was to identify the gene responsible for enlarged ears in pig.

**Results:**

First, we narrowed down the QTL region on SSC5 to a 137.85-kb interval that harbors only the *methionine sulfoxide reductase B3* (*MSRB3*) gene. Then, we identified a 38.7-kb copy number variation (CNV) that affects the last two exons of *MSRB3* and could be the candidate causative mutation for this QTL. This CNV showed complete concordance with genotype at the QTL of the founder animals in a white Duroc × Erhualian F_2_ intercross and was found only in pigs from six Chinese indigenous breeds with large ears and from the Landrace breed with half-floppy ears. Moreover, it accounted for the significant association with ear size on SSC5 across the five pig populations tested. eQTL mapping revealed that this CNV was significantly associated with the expression of the microRNA (miRNA) miR-584-5p, which interacts with *MSRB3*, one of its target genes. In vivo and in vitro experiments confirmed that miR-584-5p inhibits the translation of MSRB3 mRNA. Taken together, these results led us to conclude that presence of the 38.7-kb CNV in the genome of some pig breeds affects ear size by altering the expression of miR-584-5p, which consequently hinders the expression of one of its target genes (e.g. *MSRB3*).

**Conclusions:**

Our findings shed insight into the underlying mechanism of development of external ears in mammals and contribute to a better understanding of how the presence of CNV can regulate gene expression.

**Electronic supplementary material:**

The online version of this article (10.1186/s12711-018-0442-6) contains supplementary material, which is available to authorized users.

## Background

The size and type of ears are important conformation characteristics that distinguish pig breeds. Many Chinese indigenous pig breeds, such as Erhualian and Min, show unusually large and floppy ears. In contrast, Wild boars and European commercial breeds such as Large White and Duroc have small and erect ears. In humans, congenital underdevelopment of the external ear or microtia affects about 0.83 to 17.40 newborns per 10,000 in different ethnic populations [1]. The pig is an important biomedical model [2] and naturally occurring mutations that affect ear size in pigs offer opportunities to investigate the mechanisms that underlie development of human external ears. To date, quantitative trait loci (QTL) for porcine ear size have been identified on several pig chromosomes (SSC for *Sus scrofa*) [3–5], of which two major QTL have consistently been detected on SSC5 and 7 in multiple populations [5–7]. Previously, we showed that a missense mutation in the *peroxisome proliferator activated receptor delta* (*PPARD*) gene was the causative mutation for the QTL on SSC7 [8]. For the QTL on SSC5, Zhang et al. [9] analyzed the association of two SNPs within the *methionine sulfoxide reductase B3* (*MSRB3*) gene with ear size in a Large white × Min F_2_ intercross. We refined the QTL on SSC5 to a 8.7-cM interval and proposed *HMGA2* as a candidate gene [7]. Interestingly, the genomic region between 31.74 and 33.78 Mb on SSC5 shows a high level of genetic differentiation between breeds with floppy ears and breeds with prick/partly floppy ears [10]. In addition, this region is orthologous to a QTL for ear type in dogs [11–13] and a strong selection signature within *MSRB3* has been reported in large-eared sheep [14]. However, the causative mutation(s) for this QTL remain(s) unexplored in mammals.

In this paper, we report (1) the identification of a 38.7-kb copy number variation (CNV), which involves part of *MSRB3*, as the suggestive causative mutation for this QTL on SSC5, and (2) the underlying biological mechanism that is responsible for its effect on ear size.

## Methods

### Animals and measurement of the ear phenotype

Five pig populations that included 912 White Duroc × Erhualian F_2_ pigs (hereafter referred to as F_2_), 403 Sutai (F_19_ of a Duroc × Meishan cross), 312 purebred Laiwu, 343 Duroc × (Landrace × Yorkshire) (DLY), and 331 purebred Erhualian pigs were phenotyped and used for genome-wide association studies (GWAS) of ear size. The F_2_ intercross was constructed and managed as described previously [6, 15]. Briefly, two White Duroc boars and 17 Erhualian sows were mated to produce F_1_ animals, and then nine F_1_ sires and 59 F_1_ dams were randomly intercrossed to generate 1912 F_2_ individuals. A total of 912 F_2_ individuals were phenotyped after slaughter at 240 ± 3 days of age. Sutai is a Chinese synthetic breed that was originally produced by intercrossing Chinese Taihu and Western Duroc [16]. In this study, 435 Sutai pigs were generated by crossing four Sutai boars with 55 Sutai sows and slaughtered at 240 ± 3 days of age. Except for the DLY population that was raised on a commercial farm (Xiushui, Jiangxi) until slaughter (90–100 kg), the four other populations were raised on an experimental farm at Jiangxi Agricultural University in Nanchang, Jiangxi.

One hundred and forty-four purebred Landrace pigs were phenotyped for ear size and used to evaluate the association between the identified CNV and ear size. These 144 pigs were raised on a commercial pig farm (Zhengbang, Jiangxi). To evaluate the distribution of the causative CNV, we used three Chinese indigenous purebred breeds with small and erect ears (Diannan small-ear, Tibetan and Mingguang small-ear), five Chinese indigenous purebred breeds with large and floppy ears (Hetao large-ear, Min, Mi, Jiaxing Black and Meishan), three western commercial breeds with small and erect ears (Large White, Duroc and White Duroc), for illustrations see (Additional file 1: Figure S1) and for the number of individuals per breed see Table 1, and 11 Chinese wild boars.

**Table 1:** Distribution of the 38.7-kb CNV across diverse pig breeds with different ear size

Phenotype	Breed	*n*	CNV state		
			**−/−**	**±**	**+/+**
*Chinese breeds*					
Small and erect ears	Tibetan pig	12	12	0	0
	Diannan small-ear	11	11	0	0
	Mingguan small-ear	13	13	0	0
Large and floppy ears	Erhualian	22	0	3^a^	19
	Jiaxing black	11	0	1	10
	Meishan	10	0	1	9
	Mi pig	11	0	0	11
	Min pig	17	0	0	17
	Hetao	11	3	2	6
Chinese wild boar		11	11	0	0
*Western breeds*					
Small and erect ears	Large white	10	10	0	0
	White Duroc (F_0_ boar)	2	2	0	0
	Duroc	10	10	0	0
Half floppy ears	Landrace	11	8	1	2

^a^Three heterozygotes of Erhualian were founder dams of the White Duroc × Erhualian F_2_ intercross

Two methods were used to determine ear size: (1) the ear size of 912 F_2_, 331 Erhualian, 435 Sutai, 343 DLY and 314 Laiwu pigs, was measured as previously described [6]: after dissection from the slaughtered pig, each ear profile was drawn on a sulphate paper, which was scanned, and ear areas were calculated by the Leica Qwin area measurement software (Leica, USA); and (2) for the 144 Landrace pigs, ear size was measured on live animals by taking photographs [7]: the ears of each animal were fixed and photographed with a ruler placed on the surface of the ear as an internal size reference, and the surface of each ear was calculated using the Leica Qwin software (Leica, USA). In addition, we recorded the body weight of each phenotyped pig to use as covariate in the statistical analysis.

### Collection of ear tissue samples for gene expression analysis

Ear tissue was sampled from 26 Sutai two-day old piglets, i.e. 10 CNV/CNV, 10 wild-type, and six CNV/wild-type piglets, and used for gene expression analysis. Of these 26 samples: six (three CNV/CNV and three wild-type piglets) were used for qRT-PCR, RNA sequencing, and miR-584-5p expression, western blot, and immunohistochemistry analyses; six (three CNV/CNV and three wild-type) piglets were used for Western blot and miR-584-5p expression analysis; four (two CNV/CNV and two wild-type) piglets were used for miR-584-5p expression and qRT-PCR analysis; six (CNV/wild-type piglets) were used for western blot analyses only; and the remaining four samples (two CNV/CNV and two wild-type piglets) were used for qRT-PCR analysis only. Furthermore, ear tissue samples from 59 Landrace piglets were used for eQTL mapping of miR-584-5p. Storage of ear tissue samples differed depending on their future use, i.e. for RNA-based expression analysis, samples were stored in RNAlater (Ambion, UK) at − 80 °C until use, for Western blot analysis, samples were immersed in liquid nitrogen and then stored at − 80 °C until use, and for immunohistochemistry analysis, samples were embedded and frozen in OCT freezing medium (Tissue-Tek, Japan).

### SNP genotyping

Genomic DNA was extracted from ear tissue by the standard phenol/chloroform method. DNA quality was evaluated using a Nanodrop-1000 spectrophotometer (Thermo Fisher, USA). The F_2_, Sutai, Laiwu, Erhualian and DLY populations were genotyped for 62,163 single nucleotide polymorphisms (SNPs) on the porcine SNP 60 K Beadchip (v1.0) (Illumina, USA) according to the manufacturer’s protocol. Quality control of SNP genotype data was performed separately for each population by the check marker function of GenABEL [17]. SNPs with a call rate higher than 95%, a minor allele frequency lower than 1% and a *P* value for deviation from Hardy–Weinberg equilibrium (HWE) less than 10^−6^ as well as X-linked SNPs that were likely autosomal (odds > 1000) were removed from further analyses. Animals with a SNP call rate lower than 95% were also excluded.

### GWAS

First, we performed a GWAS of ear size for each of the F_2_, Sutai, DLY, Laiwu and Erhualian populations. Associations of SNPs with ear size were evaluated using a mixed linear model that included a random polygenic effect with the variance–covariance matrix proportional to genome-wide identity-by-state derived using SNP genotypes [18] to account for population stratification. Sex and batch were included as fixed effects, and carcass weight was treated as a covariate. The single-marker GWAS was performed using the GenABEL package [17, 19]. Presence of residual population stratification was evaluated by examining the distribution of test statistics generated from thousands of association tests and assessing their deviation from the null distribution in a quantile–quantile (Q–Q) plot [20], which was created using the R software.

A meta-analysis of the GWAS for the five populations was conducted by summing, for each SNP, the statistical Chi square (*χ*^*2*^) value of the SNP for each experimental population to calculate a new *χ*^*2*^ value with four degrees of freedom. Bonferroni correction was used to adjust for multiple-testing by using a threshold *P* < 0.05 divided by the number of SNPs used for GWAS analysis as a genome-wide significance level (see Additional file 2: Table S1). Linkage disequilibrium (*r*^2^) between SNPs within the QTL region was estimated by PLINK (v1.07) [21]. To determine each confidence interval, a region was defined with all SNPs (*r*^2^ > 0.8) in strong LD with the peak SNP. Then, within that region, the confidence interval was defined by the 2-LOD drop-off method, i.e. all SNPs with a lod-score higher than the peak lod score minus 2 were retained [22]. The phenotypic variances explained by the top SNPs were calculated by (V_reduce_ − V_full_)/V_reduce_, where V_full_ and V_reduce_ are residual variances of the models for the association analyses with and without the effect of the top SNP genotype, respectively.

### Haplotype sharing analysis

Haplotypes were reconstructed with the dualPHASE software [23] for all F_0_, F_1_ and F_2_ animals in the F_2_ population using the SNPs on SSC5. Phenotypic differences between F_2_ animals that carried different haplotypes were compared by applying the *t*-test in the lm function of R as described previously [23]. The QTL genotypes of the 19 F_0_ animals were inferred by integrating the analysis of the segregation of phenotypic values and haplotypes in the F_2_ pigs. To further identify the shared haplotype within the QTL region in large-eared pig breeds, haplotype sharing analysis was also performed between Erhualian and Min pigs using whole-genome resequencing data, as described in our previous study [24]. We reconstructed the haplotypes within a 0.99 Mb region, which covers the QTL intervals on SSC5 that were detected in the GWAS in four Erhualian and six Min pigs using 118 SNPs from this resequencing data. Wuzhishan (n = 3) and Tibetan (n = 3) pigs with small and erect ears were used as controls using the whole-genome resequencing dataset [24].

### Whole-genome sequencing

The genomes of the 19 F_0_ pigs of the F_2_ population were resequenced at ~ 25 to 30X coverage on an Illumina Hiseq 2500 platform, as described previously [24]. Clean reads were aligned to the Sscrofa11.1 pig reference genome by using the Burrows-Wheeler alignment software [25]. Local realignment around potential insertions/deletions (InDel) and base quality recalibration were conducted with the GATK software [26]. Variants within the QTL interval on SSC5 were called using the HaplotypeCaller algorithm implemented in GATK [26]. Raw SNPs were filtered with the GATK Variant Filtration tool using the following criteria: QD < 2.0, FS > 60.0, MQ < 40.0, a haplotype score > 13.0, mapping quality rank sum < -12.5 and ReadPosRankSum < − 8.0. Raw InDel were filtered by setting the following criteria QD < 2.0, FS > 200.0 and ReadPosRankSum < − 20.0. CNV were called using the depth of coverage method described by Nord et al. [27].. Briefly, first, sequence coverage was normalized and corrected for capture bias associated with GC-content; then a sliding window was used to identify regions for which the majority of bases had a coverage ratio ≥ 1.4 or ≤ 0.6, which indicates a copy number gain or loss, respectively. A 20-bp window with a minimum of 18 bp meeting the criteria for either gain or loss was defined as a variant window. CNV regions were generated by extending variant windows and merging neighboring variant regions. CNV were examined for presence of partially mapped reads within the predicted breakpoint region.

### Determination of the breakpoints of the causative CNV

The breakpoints of the 38.7-kb CNV were determined by PCR and Sanger sequencing with the primers 5′-CGAGGGAGTAAGGCAGACAG-3′ and 5′-GGCTCGGATCATCAGTATCG-3′. The PCR reaction contained 1 × buffer, 2 mM Mg^2+^, 2 mM dNTP, 200 nM of each primer, 2.5U rTaq polymerase (Takara, Japan), and 50 ng genomic DNA, and thermocycling conditions were as follows: 94 °C for 4 min, 40 cycles (94 °C for 30 s, 68–55 °C touchdown for 30 s, 72 °C for 1 min) and a final step at 72 °C for 10 min. PCR products were run on 1.5% agarose gels and sequenced after purification with the PCR products purification kit (Qiagen, Germany).

### CNV genotyping

The number of 38.7-kb CNV was estimated using both the comparative C_t_ (2^−ΔΔCt^) relative quantification (qPCR) and the absolute quantification method (ddPCR for droplet digital PCR). In total, 435 Sutai, 314 Laiwu, 343 DLY, and 144 Landrace pigs were genotyped for the causative CNV by qPCR. Briefly, one pair of primers and a probe (MSRB3-CNV1) (see Additional file 3: Table S2) were designed with the Primer Express 3.0 software (Applied Biosystems, USA). The 20-μL-reaction system contained 1 × Premix Ex Taq (TaKaRa, China), 1 × ROX Reference Dye II (TaKaRa, China), 20 nM FAM-labeled MGB probe (Applied Biosystems, USA), 4 pmol each of the forward and reverse primers and 21.5 ng genomic DNA. The *glyceraldehyde*-*3*-*phosphate dehydrogenase* (*GAPDH*) gene was used as an internal control. Real-time PCR was performed in a 7500 fast real-time PCR system instrument (Applied Biosystems, USA). Each sample was analyzed in triplicate. In order to validate the accuracy of genotyping, some samples were randomly drawn and genotyped by another pair of primers and probe using the same reaction system and parameters (MSRB3-CNV2) (see Additional file 3: Table S2).

The number of CNV in 12 samples from six large-eared pig breeds was determined by ddPCR, as described previously [28, 29]. Briefly, genomic DNA was digested with the restriction enzyme *Bcu*I (NEB, UK) that cuts the DNA outside of the amplicons. Primers and TaqMan probes used for ddPCR were the same as those used for the qPCR (MSRB3-CNV1) (see Additional file 3: Table S2). The *estrogen receptor* 1 (*ESR1*) gene was used as reference and was labeled with VIC at the 5′ end. The ddPCR reaction mixture was prepared in a 22-μL volume containing 40 ng of digested DNA template, 1 × ddPCR master mix (Bio-Rad, USA), 1.1 μL of each target primer (0.9 μM) and probe (0.25 μM) mix, and 1.1 μL of each reference primer (0.9 μM) and probe (0.25 μM) mix. The disposable cartridge was loaded with the reaction mixture and droplet generation oil and then placed into a QX100 Droplet Generator (Bio-Rad, USA) to acquire droplets. The droplets were run on a Thermal Cycler (Bio-Rad, USA) as follows: 95 °C for 10 min, 40 cycles (94 °C for 30 s, 60 °C for 1 min), 98 °C for 10 min and then kept at 12 °C. After amplification, we loaded the plate on the QX100 Droplet Reader (Bio-Rad, USA) to read the droplets. The Droplet reader processed each sample independently and interrogated both FAM and VIC fluorescence under the guidance set in the Quanta soft software (V1.3.2.0). The software measured the numbers of positive and negative droplets per fluorophore per sample, which were converted into digital signals. The numbers of CNV copies in the tested samples were read directly from the software (Bio-Rad, USA).

### Association and interaction analyses

Association of the CNV with ear size was tested using a general linear mixed model [18]:

$$y = u + gender_{j} + batch_{i} + cw + CNV + e,$$

where *y* is the phenotype of ear size, *u* is the intercept of phenotype after correction for sex, batch and carcass weight, *gender*_*j*_ is the sex (j = 0, 1) and *batch*_*i*_ is the slaughter batch (i = 1, 2, 3, 4, …), which were included as fixed effects, *cw* is the carcass weight that was treated as a covariate, *CNV* represents the effect of the CNV on ear size, which was estimated by treating the relative quantity (RQ) values as quantitative covariates, and *e* is the residual. The analysis was conducted by using the GenABEL package in R software [17].

A similar model was used to test the interaction between the two causative mutations (*PPARD* G32E and the *MSRB3* 38.7-kb CNV) for ear size:

$$y = \mu + gender_{j} + batch_{i} + cw + PPARD + MSRB3 + PPARD \times MSRB3 + e,$$

where *PPARD* indicates the effect of *PPARD* G32E on SSC7 as a fixed factor, *MSRB3* indicates the effect of the *MSRB3* 38.7-kb CNV on SSC5 as a fixed factor, and *PPARD* × *MSRB3* is the interaction effect between the two variants as a fixed factor.

### qRT-PCR

Ear tissue samples from 10 CNV/CNV and 10 wild-type Sutai piglets were used for qRT-PCR analysis (as described above). Total RNA was extracted using the Rneasy Fibrous Tissue mini kit (Qiagen, Germany). The first-strand cDNA was synthesized from 2 μg of total RNA using the Omniscript reverse transcriptase kit (Qiagen, Germany). Expression profiles of the two transcript isoforms of *MSRB3* were determined using the SYBR Green method. qRT-PCR was performed in a 20-μL reaction volume containing 50 ng of template cDNA, 1 × Power SYBR Green Master mix (Applied Biosystems, USA) and 5 pM of each primer, with the following thermocycling conditions: 15 min at 95 °C and 48 cycles (95 °C for 15 s, 60 °C for 1 min and 72 °C for 1 min). Then, a dissociation curve analysis was performed. Expression levels of *HMGA2* and *LEMD3* were determined by the Taqman method. The 20-μL reaction volume included 50 ng of template cDNA, 1 × Premix Ex Taq (TaKaRa, China), 1 × ROX Reference Dye II (TaKaRa, China), 4 pM of each primer and 2 nM of probe, with the following thermocycling conditions:15 min at 95 °C and 40 cycles (95 °C for 15 s and 60 °C for 1 min). *β*-*actin* was used as an internal control. Gene-specific primers and probes were designed using the primer premier 5.0 software (see Additional file 4: Table S3). qRT-PCR was performed using the 7500 Fast real-time PCR System (Applied Biosystems, USA). Each sample was analyzed in triplicate.

### RNA sequencing

RNA sequencing was done on ear tissue from six Sutai piglets, including three CNV/CNV and three wild-type animals. DNA contamination was removed from total RNA by incubation with RNase-free DNase I (NEB, UK) at 37 °C for 30 min. The quality of total RNA was assessed by a 2100 Bioanalyzer (Agilent, USA) and 1% agarose gel electrophoresis. RNA sequencing was performed as described previously [30]. Briefly, mRNA was isolated from total RNA with oligo (dT) magnetic beads (Invitrogen, USA) and then fragmented using the RNA fragmentation kit (Ambion, USA). The first-strand cDNA was synthesized using random hexamer primers and reverse transcriptase (Invitrogen, USA), while the second-strand cDNA was synthesized using RNase H (Invitrogen, USA) and DNA polymerase I (NEB, UK). The cDNA libraries were loaded onto flow cell channels of a HiSeq 2000 platform (Illumina, USA) for paired-end 90 bp × 2 sequencing. The average insert size for the paired-end libraries was 200 bp. A paired-end cDNA library was constructed for each of the six samples.

The raw sequence dataset was processed to produce clean sequence data, which were then aligned to the porcine reference genome sequence (Sscrofa 11.1) using TopHat [31]. The gene expression value was determined and normalized using fragments per kilobase of exon per million fragments mapped (FPKM) [32]. The Cuffdiff program in Cufflinks [33] was used to identify differential expression genes (DEG) based on |log_2_ (FC)| > 1, where FC is fold change, and q-value (an adjusted *P* value) < 0.01. Potential alternative splicing transcripts of the *MSRB3* gene were estimated in all six sequenced samples using the Cuffdiff program. We refined the gene structure by assembling transcripts with clean reads with the Cufflinks software [34]. The assembled transcripts were compared to gene annotations from the reference genome to detect the extensions at the 5′ and 3′ ends of the corresponding gene annotation. In this study, we focused on the 3′ gene boundary of *MSRB3*.

### Western blot

Total protein was extracted from ear tissue samples of 18 Sutai piglets (described above) and porcine fetal fibroblast (PFF) cells by using the total protein extraction kit (Applygen, China) and quantified with the BCA Protein Quantification kit (Vazyme, China). Then, the extracted proteins were separated by SDS-PAGE. After transfer, the nitrocellulose membranes were incubated with rabbit anti-MSRB3 primary antibody (orb373913, Biorbyt, UK) at a 1:400 dilution and mouse anti-β-actin primary antibody (A1978, as endogenous control) (Sigma, USA) at a 1:1000 dilution. After incubation with secondary antibodies (goat anti-rabbit IgG peroxidase antibody (A0545) and rabbit anti-mouse IgG peroxidase antibody (A9044) for MSRB3 and β-actin, respectively) that were conjugated with horseradish peroxidase (Sigma, USA), the nitrocellulose membranes were visualized by using a BeyoECL plus kit (Beyotime Biotechnology, China). The images were acquired with the GeneSnap software (SynGene, UK) and quantified by the ImageJ software [35].

### Immunohistochemistry

Ear tissue samples from six Sutai piglets were used for immunohistochemistry analysis (described above). All ear tissue samples that had been embedded and frozen in OCT freezing medium (Tissue-Tek, Japan) were cut in sections of 4 μm with a cryostat. The sections were fixed in 4% paraformaldehyde in phosphate buffered saline (PBS) for 10 min at room temperature, and then permeated in PBS containing 0.2% Triton X-100. The sections were blocked for 30 min in PBS containing 10% bovine serum albumin and 0.3% Triton X-100, and then incubated overnight at 4 °C in blocking solution containing the primary antibodies (orb373913, Biorbyt, UK) at a 1:100 dilution. The secondary antibody, goat anti-Rabbit IgG (whole molecule) (F9887, Sigma, USA) conjugated with fluorescein isothiocyanate at a dilution, was used to incubate the slides at room temperature for 45 min. Immunohistochemistry images were captured using a Nikon ECLIPSE Ti inverted microscope equipped with NIS-Elements D software (Nikon, Japan).

### Expression profiling analysis of miR-584-5p in vivo and eQTL mapping

The mature miRNA-584-5p sequence was predicted by using the miRBase database (release 20) [36]. Expression profiles of miR-584-5p in ear tissue from eight CNV/CNV and eight wild-type Sutai piglets, along with 59 Landrace piglets were analyzed. Extraction and quality control of total RNA were performed as described above. A stem-loop RT-PCR method [37] was used to design the primers and probes to quantify the expression level of miR-584-5p using the 2^−ΔΔCt^ method (see Additional file 5: Table S4). The *U6* gene was chosen as endogenous control. The qRT-PCR reaction and conditions were the same as those described above.

eQTL mapping was performed for miR-584-5p in ear tissue samples from 59 Landrace two-day old piglets. Thirty-five SNPs within the QTL interval on SSC5 were genotyped by PCR amplification and Sanger sequencing. CNV genotypes were determined by qPCR on an ABI7900HT instrument (Applied Biosystems, USA), as described above. The expression values of miR-584-5p were adjusted for the effects of sex, batch, and kinship using a robust linear regression model [38]. eQTL mapping was performed using a mixed linear model implemented by the *mmscore* function of GenABEL in R package. Bonferroni correction was applied to adjust for multiple-testing.

### In silico prediction of the target genes of miR-584-5p

The DIANA-microT web server (v5.0) was used to predict the target genes of miR-584-5p based on the rule that the miRNA regulate gene expression mainly by base pairing with miRNA-recognition elements (MRE) in the target mRNA 3′UTR or coding sequences (CDS) [39]. The miTG (miRNA targeted genes) score was calculated by microT-CDS using the weighted sum of the scores of all conserved and non-conserved MRE in the 3′UTR and CDS of the target mRNA. The higher the score, the higher the probability of targeting MRE that are within the 3′UTR or in the CDS of the mRNA.

### Construction of plasmids and luciferase reporter assay

The 3′UTR of *MSRB3* was synthesized and cloned into the vector downstream of the firefly luciferase ORF by cloneEZ (Genscript, China). We obtained a pGL3 luciferase reporter with the mutant 3′UTR of *MSRB3* that has a 7-bp deletion in the target site. Porcine fetal fibroblast (PFF) cells were co-transfected with 100 ng of the wild-type or mutant 3′UTR luciferase reporter and 100 nM of the miR-584-5p mimics or the negative control duplexes using lipofectamine 3000 (Invitrogen, USA) in 24-well plates. Each treatment was replicated three times. After transfection for 48 h, cells were harvested by adding 200 μL of passive lysis buffer. Renilla and firefly luciferase activities were assayed on an Infinite 200 PRO multimode reader (Tecan, Switzerland) using the luciferase assay system from Promega. The relative luciferase activity was normalized to the Relina luciferase activity in each sample. We compared the normalized firefly luciferase activity between the wild-type and mutant reporters using the Student’s t test (*P* < 0.05). This assay was repeated in triplicate.

### Transfection of the miR-584-5p mimic to PFF cells

PFF cells (2 × 10^5^ cells seeded in 6-well plates) were cultured in DMEM (with high glucose and pyruvate) (Gibco, USA) containing 10% fetal bovine serum, 50 U/mL penicillin and 50 μg/mL streptomycin (Gibco, USA) and incubated in a humidified incubator with 5% CO_2_ at 37 °C. When the cells were 70% confluent, the mirVana miRNA Mimic negative control (Ambion, USA) and the miR-584-5p mimic (Ambion, USA, 5′-UUAUGGUUUGCCUGGGACUGAG-3′) were transfected into the PFF cells with 6 μL of 100 nM lipofectamine 3000 (Invitrogen, USA). After 7 h, the transfection mix was replaced by complete medium. Cells were harvested 24 h after transfection. Total proteins were extracted from the cultured cells and used for western blot analysis using the protocol described above.

## Results

### GWAS in diverse populations confirms the QTL for ear size on SSC5

**Fig. 1:**
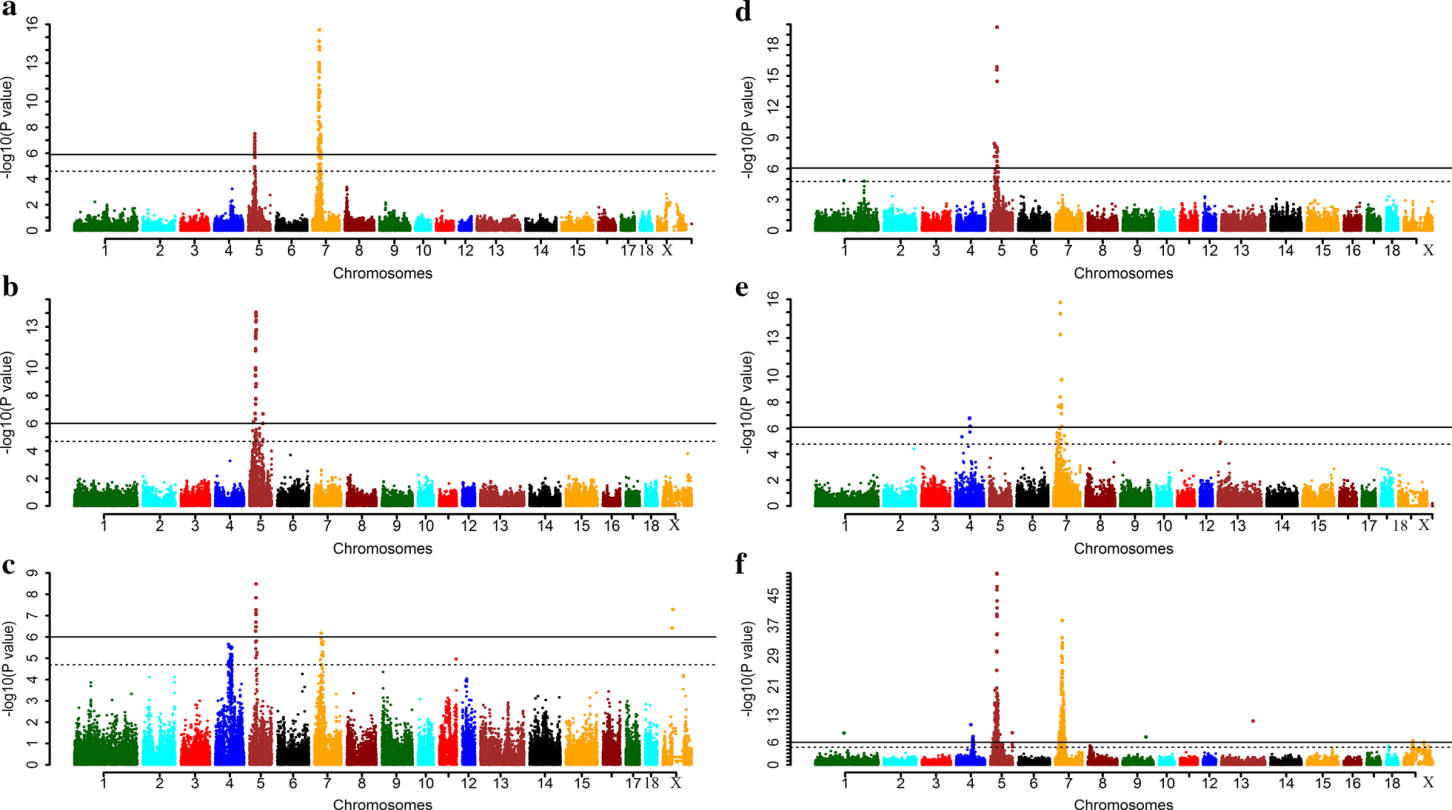
Manhattan plots of genome-wide association studies to map a major QTL for porcine ear size. GWAS were performed in the White Duroc × Erhualian F_2_ resource population (**a**), Sutai (**b**), Laiwu (**c**), Duroc × Landrace × Yorkshire (**d**), and Erhualian pig populations (**e**). A meta-analysis of the GWAS was also performed across these five populations (**f**). In the Manhattan plots, −log _10_ (*P* values) of the SNPs are plotted against their positions on the porcine chromosomes, with chromosomes shown in different colors for clarity. The solid line indicates the 5% genome-wide significance threshold and the dashed line shows the suggestive-significance threshold (5% chromosome-wide significance)

To narrow down the interval on SSC5 that harbors the QTL for ear size, we performed GWAS in five populations, totaling 2301 pigs. For all these pigs, detailed records on ear size and genotypes for ~ 62,000 SNPs from the Illumina porcine 60 K SNP chip were available. The final set of informative SNPs for GWAS in each population is in Additional file 2: Table S1. First, we conducted a GWAS in each of the five pig populations using a linear regression model assuming an additive effect (see “Methods”). The inflation factor (λ) values for genomic control [40] ranged from 0.96 to 1.08, which indicates that there was no population stratification in our data (see Additional file 6: Figure S2). We identified genome-wide significant association signals on SSC4, 5, 7 and X (Fig. 1). In the F_2_ and Erhualian populations, the top SNPs were located around the *PPARD* gene on SSC7 (30.14 Mb, LOD > 15.58). The second most significant signal was observed at 29.94 Mb on SSC5 in the F_2_ population (ALGA0031524, LOD = 7.50) (Fig. 1). No association signal was observed on SSC5 in the Erhualian population. For the Sutai and the DLY populations, we detected a genome-wide significant QTL only on SSC5 and the most significant SNPs were detected on SSC5 at 29.94 Mb (ALGA0031524, LOD = 14.05) and 29.69 Mb (ASGA0025245, LOD = 19.72), respectively. In Laiwu pigs, the strongest signal was also observed on SSC5, between 29.55 and 30.07 Mb, a region that harbored four genome-wide significant SNPs: ASGA0025241, ASGA0025245, H3GA0016181 and ALGA0031527 (LOD = 8.49), while other genome-wide significant SNPs were identified on SSC4, 7 and X (LOD > 6.00). The top SNPs on SSC5 explained 11.5, 27.4, 12.4 and 27.9% of the phenotypic variance for ear size in the F_2_, Sutai, Laiwu and DLY populations, respectively. Then, we performed a meta-analysis of the GWAS across the five pig populations, which identified the most significant SNP on SSC5 at 29.69 Mb (ASGA0025245, LOD = 50.90, Fig. 1).

**Fig. 2:**
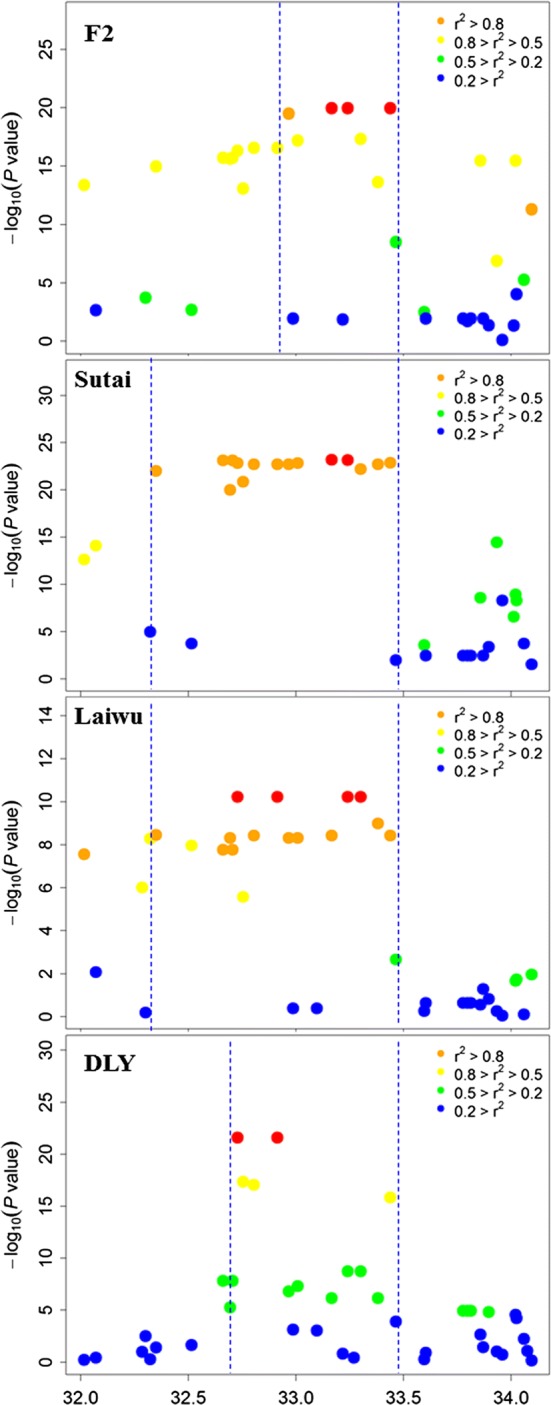
Regional Manhattan plots for association analyses around the QTL for ear size on chromosome 5 in four pig populations. The top SNPs are highlighted by red dots. Different levels of linkage disequilibrium (LD based on *r*^*2*^) of the top SNPs with surrounding SNPs are indicated by different colors. The QTL intervals indicated by dashed lines were defined by the 2 drop-off of LOD value and *r*^*2*^ ≥ 0.8. Their overlapping region spans 509.28 kb between 29.74 and 30.25 Mb on chromosome 5

To determine the empirical confidence interval of this QTL, we identified SNPs that were in the regions around the top SNPs on SSC5 that had linkage disequilibrium (*r*^*2*^) values with the top SNP higher than 0.8. To correct for potential effects at other loci, we re-conducted GWAS in the F_2_ and Laiwu populations by including the *PPARD* causative mutation (G32E) on SSC7 and the top SNPs on SSC4 and X as fixed effects in the linear regression model. By applying the 2-LOD drop-off and *r*^*2*^ ≥ 0.8 criteria (see “Methods”), we estimated the empirical confidence intervals for the QTL on SSC5 in the F_2_, Sutai, Laiwu and DLY populations at 509.28 kb (29.74–30.25 Mb), 894.37 kb (29.36–30.25 Mb), 921.14 kb (29.33–30.25 Mb) and 696.68 kb (29.55–30.25 Mb), respectively (Fig. 2). The smallest confidence interval (509.28 kb) in the F_2_ population could be due to recombination within the interval that led to the segregation of the QTL on SSC5 in the founder dams (see below). Thus, this is the most likely QTL interval.

### Haplotype-sharing analysis refined the 509.28-kb QTL interval on SSC5 to 137.85 kb

**Fig. 3:**
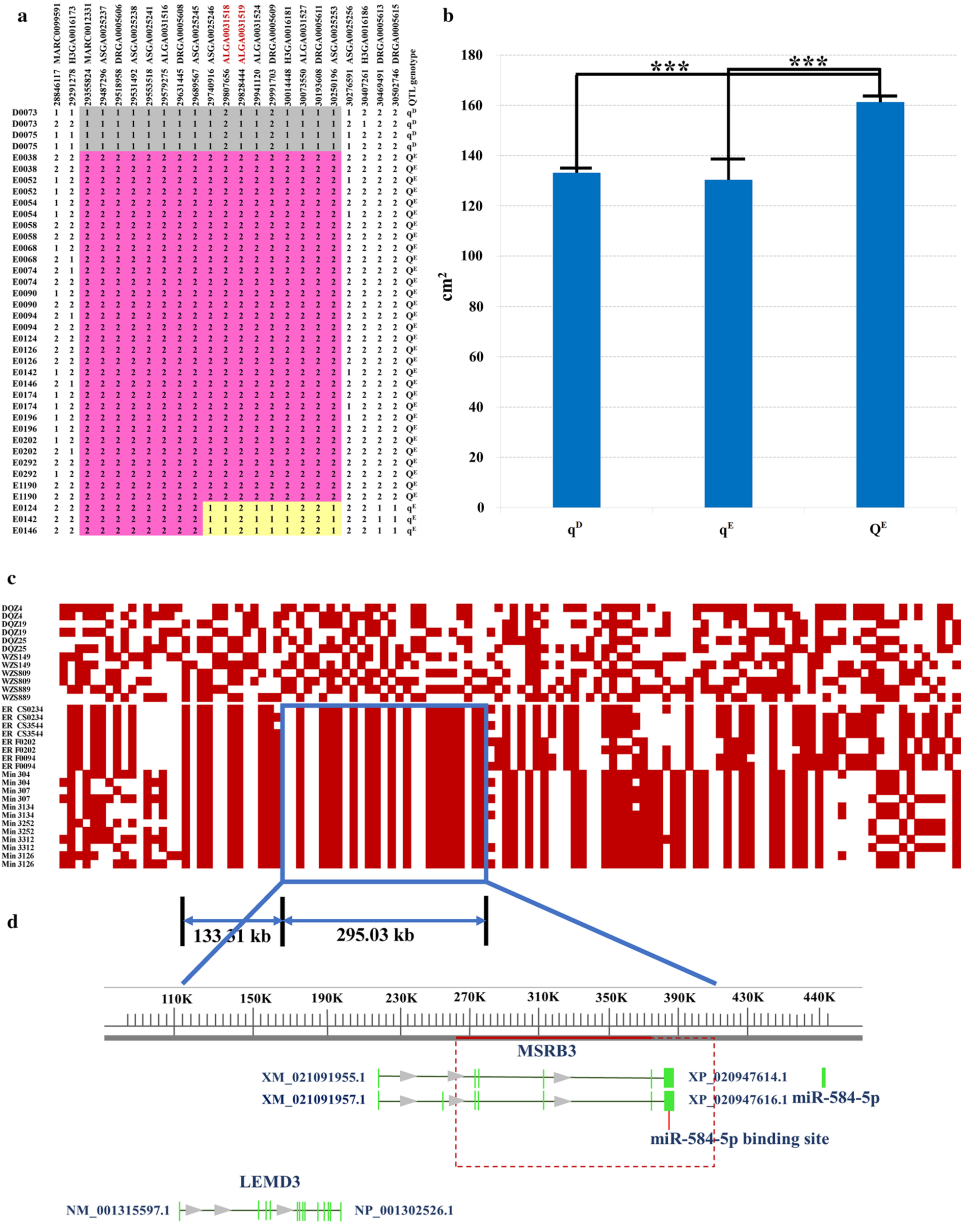
Haplotype-sharing analysis refines the critical QTL region to a 137.85 kb interval. **a** Haplotype-sharing analysis in the 19 F_0_ founder animals of the White Duroc × Erhualian F_2_ resource population. The SNPs and their positions are displayed at the top of the figure. For SNPs, alleles with the highest frequencies are denoted 1, while those with the lowest frequencies are denoted 2. The shared haplotypes are indicated by shaded boxes. Identities of these F_0_ animals are given on the left axis. D and E represent Duroc and Erhualian, respectively. The QTL genotype for each chromosome is shown on the right axis. The red colored box represents the shared Q-haplotype block in Erhualian sows, Individuals E0124, E0142 and E0146 carried a distinct haplotype that is marked in yellow. **b** Effects of haplotypes q^D^, Q^E^ and q^E^ on ear size in the White Duroc × Erhualian intercross (least square mean ± s.e., cm^2^). The haplotype Q^E^ significantly increased ear size. **c** Haplotype-sharing analysis in pigs with different ear sizes. The upper panel shows haplotypes of small-eared Tibetan and Wuzhishan pigs that were selected as controls. The lower panel depicts haplotypes of Min and Erhualian pigs with large and floppy ears. The sample ID is shown in the left column and the SNP ID is shown in the top row. The Min and Erhualian pigs shared two haplotypes of 133.31 and 295.03 kb, which are not found in small-eared pigs. **d** The most likely critical interval of this QTL was defined as the 137.85-kb region indicated by a red box. Exons 3–7 of *MSRB3* are located in this region. The gray arrows indicate the direction of the transcripts and the green vertical bars show the exons of the transcripts

We reconstructed the haplotypes of two founder sires and 17 founder dams in the F_2_ population by using SNPs on SSC5. Fourteen of the Erhualian founder dams shared a hapotype of ~ 0.99 Mb around the most significant SNPs (Q^E^, Fig. 3a). As predicted, this shared haplotype was associated with increased ear size and presumably with the chromosomes carrying the QTL allele increasing ear size (Q-bearing chromosomes). Three Erhualian founder sows (E0124, E0142 and E0146, Fig. 3a) carried a distinct haplotype that was denoted as q^E^. This haplotype appeared to have originated from Q^E^ with a recombination breakpoint occurring between SNPs ASGA0025245 (29.69 Mb) and ASGA0025246 (29.74 Mb) (see Fig. 3a). Two White Duroc founder sires carried another distinct haplotype q^D^. Both q^D^ and q^E^ haplotypes were associated with decreased ear size, which is different from the effect of the Erhualian Q-bearing chromosome (Q^E^). The least-square mean (± s.e.) of ear area for individuals carrying the Q^E^ haplotype was 161.26 ± 1.77 cm^2^, which is significantly larger than least square means for q^D^ (133.16 ± 1.48 cm^2^, *P* < 0.001) and q^E^ (130.28 ± 8.29 cm^2^, *P* < 0.001, Fig. 3b). This shared haplotype Q^E^ allowed us to refine the major QTL on SSC5 to a 0.99 Mb interval between SNPs H3GA0016173 (29.29 Mb) and ASGA0025256 (30.28 Mb).

Chinese Min pigs, such as Erhualian, also have unusually large and floppy ears, and we identified the same QTL for ear size on SSC5 in a Large White × Min F_2_ resource population [5]. This led us to assume that Min pigs carry the same causative mutation that affects ear size. Thus, we expected the presence of shared haplotypes around the causative mutation between Min and Erhualian pigs. To test this, we performed a haplotype-sharing analysis on 10 large-eared pigs (four Erhualian and six Min pigs) and six small-eared pigs (three Wuzhishan and three Tibetian pigs). We reconstructed the haplotypes of these pigs using the 118 SNPs in the 0.99-Mb QTL interval that contains the 509.28-kb critical region of the SSC5 QTL. As predicted, all large-eared pigs shared two chromosomal segments of 295.03 kb (29.58–29.88 Mb) and 133.31 kb (29.45–29.58 Mb) (Fig. 3c). One of the two segments, i.e. the 295.03-kb (29.58–29.88 Mb) segment overlapped with the 509.28-kb critical region (29.74–30.25 Mb). Thus, the 137.85-kb overlapping region (29.74–29.88 Mb) was the most likely confidence interval for the QTL on SSC5 on which we focused all further analyses. Exons 2–7 of the *MSRB3* gene are located within this 137.85-kb interval (Fig. 3d).

### Whole-genome sequencing identified a CNV within the *MSRB3* gene as a candidate causative mutation

First, we performed a comparative genomic sequence analysis (see “Methods”) of the 137.85-kb QTL interval and found that the reference genome (Sscrofa 11.1) was misassembled for this region (see Additional file 7: Figure S3). We constructed a corrected contig that was submitted to GenBank in NCBI with accession number MK028166. Then, we isolated the full-length mRNA sequence of *MSRB3* and identified two transcript isoforms that encode 188 and 183 amino acids, respectively (accession numbers: KX557289 and KX557290). Comparison of these two transcripts showed that the *MSRB3* exon 2 was absent in isoform 1 due to alternative splicing (see Additional file 8: Figure S4).

**Fig. 4:**
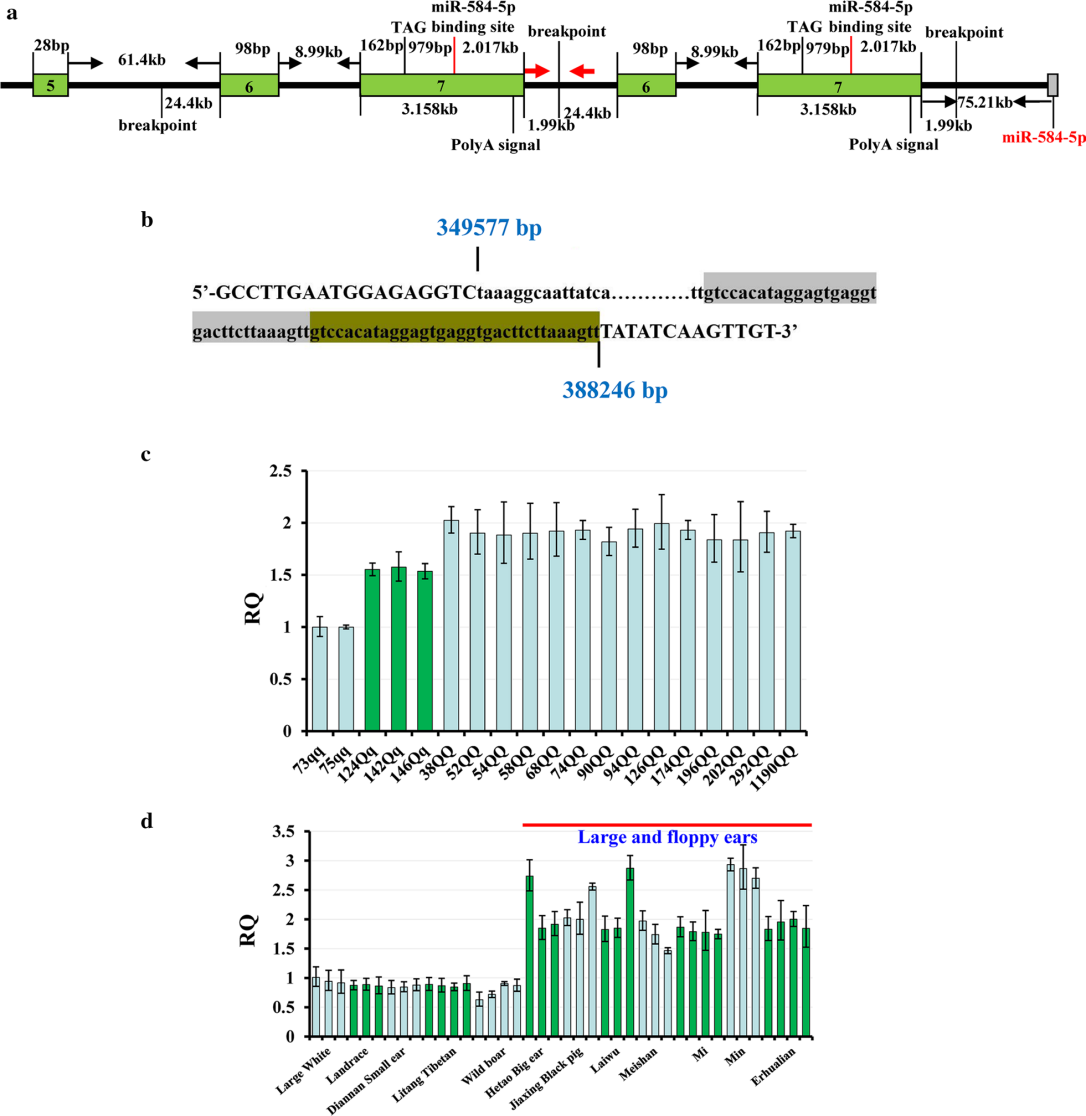
The causative CNV and its distribution in pig breeds with different ear sizes. **a** Structure of the CNV. The red arrows indicate the location of the breakpoint between two copies of the CNV and the red vertical line shows the binding site of miR-584-5p. **b** The breakpoint of the CNV. Lowercase letters represent the sequences of the CNV. A 33-bp sequence repeat at the 3′-end of the CNV is indicated by gray shading and grass green. **c** CNV distribution in the 19 F_0_ founders of the White Duroc × Erhualian F_2_ intercross. For each animal, the CNV genotypes were in complete concordance with the QTL genotypes. The *Y*-axis shows the RQ values obtained by qPCR, the *X*-axis indicates each individual’s ID and its QTL genotypes. **d** CNV distribution in pig breeds with different ear sizes. All pigs with large and floppy ears had the CNV, while none of the small-eared pigs had it

To identify the causative mutation in the QTL, we screened all candidate mutations within the 137.85 kb confidence interval using whole-genome sequencing data from the 19 F_0_ animals of the F_2_ population, for which QTL genotypes were inferred, including two *qq* (D73, D75), three *Qq* (E124, E142 and E146) and 14 *QQ* animals. The sequencing data completely covered the 137.85-kb interval and allowed us to identify 64 polymorphisms (SNPs and InDels) and one 38.7-kb CNV as candidate mutations. The 64 polymorphisms showed concordance with the genotypes at the QTL of the 19 F_0_ founder animals. However, none of these candidate mutations was within the exons of *MSRB3*. Next, we used PCR amplification and Sanger sequencing to determine the breakpoints of the 38.7-kb CNV that starts at 349,577 bp and ends at 388,246 bp of the corrected contig (MK028166) (Fig. 4a), covering the last two exons 6 and 7 of the *MSRB3* gene. We found a 33-bp sequence repeat at the 3′-end of the CNV (Fig. 4b). Among the 64 SNPs and InDel variants, 51 were located within the CNV. All 51 mutations were called as homozygous in the CNV/CNV pigs, which indicates that they were identical in each CNV copy.

In theory, the causative allele (most likely derived) for the increased ear size should not be detected or occur at a very low frequency in breeds with small and erect ears. We analyzed the allele frequencies of these 64 polymorphisms in 30 individuals from two small and erect-eared breeds (Wuzhishan and Tibetan) and in 20 individuals from two large and floppy-eared breeds (Min and Erhualian) using the dataset described in our previous publication [24]. For all small-eared pigs, the alleles that presumably increases ear size occurred at high frequencies for all 64 polymorphisms (see Additional file 9: Table S5). In contrast, the 38.7-kb CNV was absent from all small-eared pigs and present at high frequencies in large-eared pigs. This provided important supporting evidence that the 38.7-kb CNV is the most likely causative mutation for the QTL on SSC5. Next, we determined the copy number of this CNV in the 19 F_0_ founder animals of the F_2_ population (see “Methods”) and found that the genotypes of the founder animals at this CNV were completely concordant with their genotypes at the QTL, i.e. two copies in two White Duroc sires (q^D^q^D^), three copies in three Erhualian founder sows (Q^E^q^E^), and four copies in 14 Erhualian sows (Q^E^Q^E^) (Fig. 4c).

We also determined the copy number of this CNV in 12 ear tissue samples from seven large and floppy-eared pig breeds by both qPCR and ddPCR. Copy numbers in CNV/CNV homozygous Erhualian, Jiaxing Black, Laiwu, Meishan, Mi and Min pigs were equal to 4, 4, 6, 4, 4 and 6, respectively (see Additional file 10: Table S6). To investigate the distribution of this CNV in diverse pig breeds, it was genotyped in another 162 pigs from 13 breeds with distinct ear sizes. All animals from the three Chinese breeds with small and erect ears had only two copies (wild-type) at the CNV site. In contrast, nearly all animals from the six Chinese indigenous pig breeds with large and floppy ears had four copies of the CNV (Table 2, Fig. 4d). Among the four Western breeds, Large White, Duroc, and White Duroc had two copies of the wild-type allele, and three out of the 11 Landrace pigs carried more than two copies of this CNV (one carrying three copies and two carrying four copies; see Table 1). This result is in agreement with the fact that Landrace pigs have partly flopped down ears and the other three breeds have small and erect ears. Whether the causative CNV has been introgressed from Chinese large-eared pigs into Landrace pigs is not known at present and requires an evolutionary history analysis of the haplotypes around the CNV in different Chinese and Western pig breeds. We also analyzed the copy number of this CNV in 11 Chinese wild boars and found that none of these animals carried it (Table 1), which was consistent with the findings in [13] that showed that floppy ears are a derived phenotype that appeared after domestication.

**Table 2:** Association of the causative CNV with ear size in four pig populations

Population	*N* ^a^	Frequency of CNV (%)	*P* value	Phenotypic variance explained by CNV (%)	Phenotypic variance explained by top SNP (%)
Sutai	435	55.54	2.20 × 10^−16^	29.43	27.40
Laiwu	314	70.12	8.72 × 10^−10^	13.84	12.40
DLY	343	13.85	2.00 × 10^−16^	33.35	27.90
Landrace	144	42.71	1.37 × 10^−15^	32.33	–^b^

^a^*N* animal number

^b^Landrace pigs were not performed the GWAS analysis

To obtain additional supporting evidence, we performed association studies between this CNV and ear size in four pig populations, i.e. Sutai, Laiwu, DLY and Landrace. As expected, the CNV was significantly associated with ear size across the Sutai (*P* = 2.2 × 10^−16^), Laiwu (*P* = 8.72 × 10^−10^), DLY (*P* = 2.0 × 10^−16^) and Landrace pigs (*P* = 1.37 × 10^−15^) (Table 2). Compared to the top SNPs identified in the GWAS, this CNV explained more phenotypic variance for ear size in Sutai (29.4 vs. 27.4%), Laiwu (13.8 vs. 12.4%) and DLY pigs (33.4 vs. 27.9%). To further evaluate the causality of this CNV, we conducted conditional GWAS in the Sutai, Laiwu and DLY populations by including the genotypes of the CNV as a fixed effect in the GWAS statistical model. This resulted in the disappearance of the previously detected significant association signals on SSC5 across these populations (see Additional file 11: Figure S5). This finding strengthened our hypothesis that the CNV is the causative mutation underlying the QTL for ear size on SSC5.

**Fig. 5:**
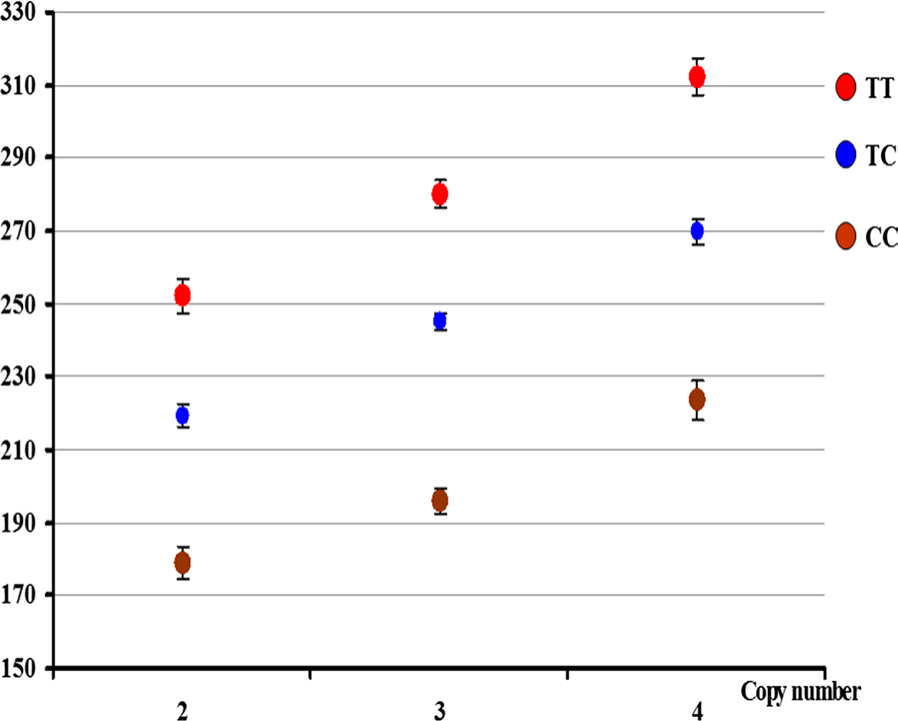
Two major QTL on SSC5 and 7 collectively affect ear size in pigs. The different color dots represent the genotypes of *PPARD* G32E (C > T), which is the causative mutation for the QTL on SSC7. The *Y*-axis indicates ear size values (cm^2^) and the *X*-axis shows the number of copies of the causative CNV for the SSC5 QTL

To test whether the two major QTL on SSC5 and 7 epistatically or additively affected ear size, we analyzed the interaction between the causative mutations (*PPARD* G32E and the 38.7-kb CNV) at the two QTL in the F_2_ population but found no significant epistatistic effect between the two mutations (Fig. 5), which suggests these two QTL have additive effects on ear size in the pig. Indeed, the two QTL collectively explained 48.2% of the phenotypic variance of ear size in the F_2_ pigs (133.5 cm^2^).

### The levels of MSRB3 in ear tissue differed significantly between small and large-eared pigs

**Fig. 6:**
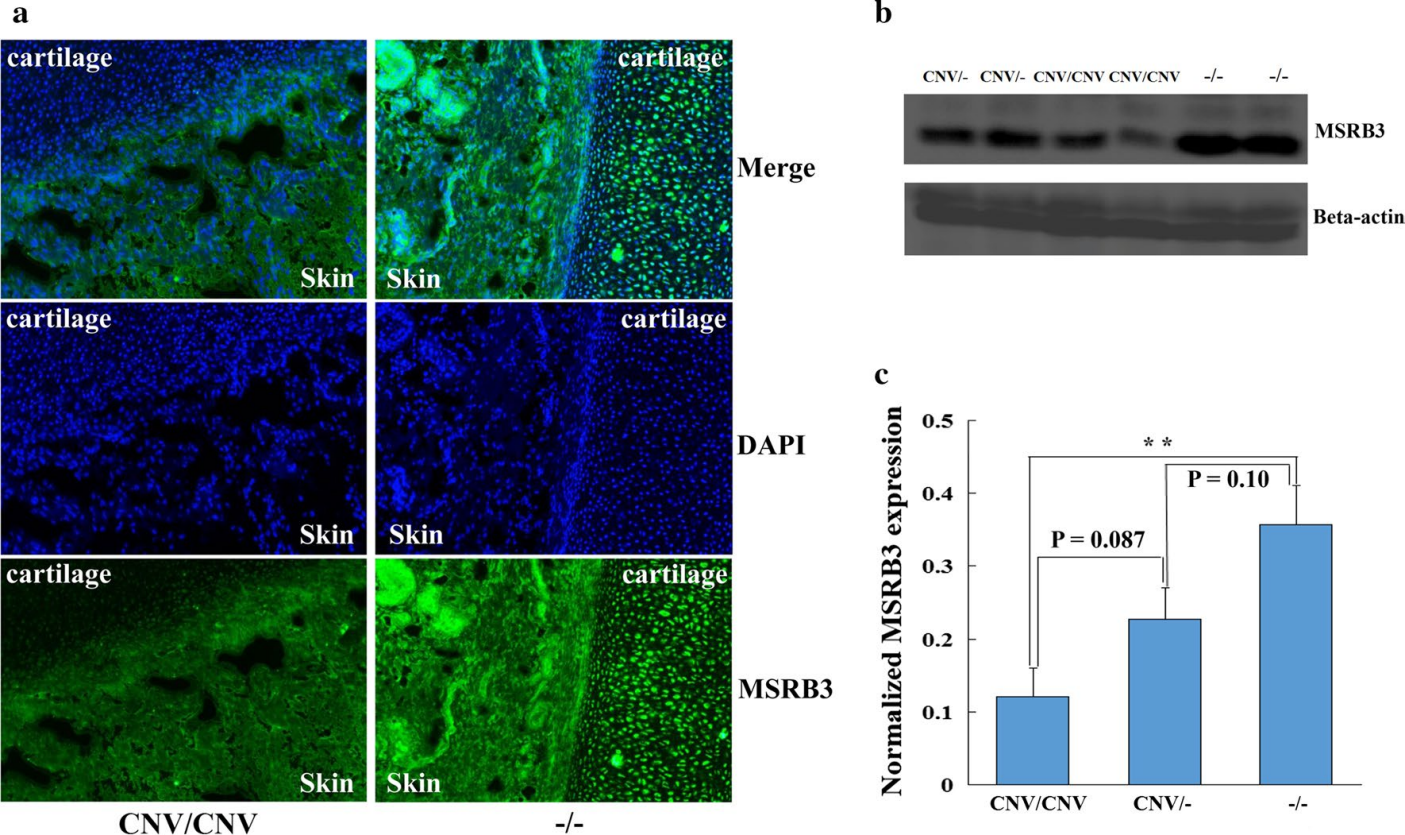
Expression of the MSRB3 protein in ear tissue samples representing different genotypes at the causative CNV. **a** MSRB3 immuno- and DAPI nuclear staining on ear tissue from wild-type (small-eared) and CNV/CNV (large-eared) piglets. The image was captured at FITC: 1 s, 20 X. A significant difference in MSRB3 abundance was found in both the skin and cartilage between CNV/CNV and wild-type pigs. **b** Western blot analysis showing MSRB3 protein levels in 18 (six CNV/CNV, six CNV/− and six −/−) pigs. The expression level of β-actin was used as loading control. **c** MSRB3 protein level in the 18 animals was quantified by the GeneTools software. The histogram illustrates the abundance of MSRB3 after normalization to β-actin abundance. **, *P* < 0.01

Because only the *MSRB3* gene is located within the 137.85-kb confidence interval, we quantified the mRNA levels of *MSRB3* and its flanking genes *LEMD3* and *HMGA2* by real-time quantitative reverse transcription PCR (qRT-PCR). Because the pinna has reached the morphology of an adult at birth [41] and the shape of the external ears (erectness or floppy) can be identified clearly, ear tissue samples from 10 CNV/CNV (large-eared) and 10 wild-type (small-eared) 2-day-old piglets were used for this analysis. Neither of the two *MSRB3* transcripts and neither of the *LEMD3* and *HMGA2* genes showed differential expression between the two groups (see Additional file 12: Figure S6). Next, we conducted a comparative RNA sequencing analysis using total RNA extracted from ear tissue of three CNV/CNV and three wild-type 2-day-old piglets (accession number: GSE80613). The structure of the three genes was investigated by aligning the reads to the reference genome and transcript assembly. We did not identify any transcript isoform for *MSRB3* that was uniquely associated with the large ear phenotype, which is most probably due to the duplicated region being located at the 3′UTR of *MSRB3*. Moreover, we did not find significant differences in the mRNA levels of *MSRB3*, *LEMD3* and *HMGA2* between the CNV/CNV and wild-type samples and we did not detect any alternative splicing events for *MSRB3* mRNA associated with ear size (see Additional file 13: Figure S7). Then, we investigated the spatial distribution of MSRB3 in the external ears of both CNV/CNV and wild-type two-day-old piglets by immunohistochemistry. MSRB3 was expressed in both the skin and cartilage of the ear, with a higher expression level in the external ear tissues from wild-type piglets (Fig. 6a). Western blot analysis revealed a higher level of MSRB3 in wild-type compared to CNV/CNV piglets (Fig. 6b, c) (see Additional file 14: Figure S8), which was consistent with the results of the immunohistochemistry analysis.

### The causative CNV affects the expression level of miR-584-5p, which suppresses the expression of MSRB3

**Fig. 7:**
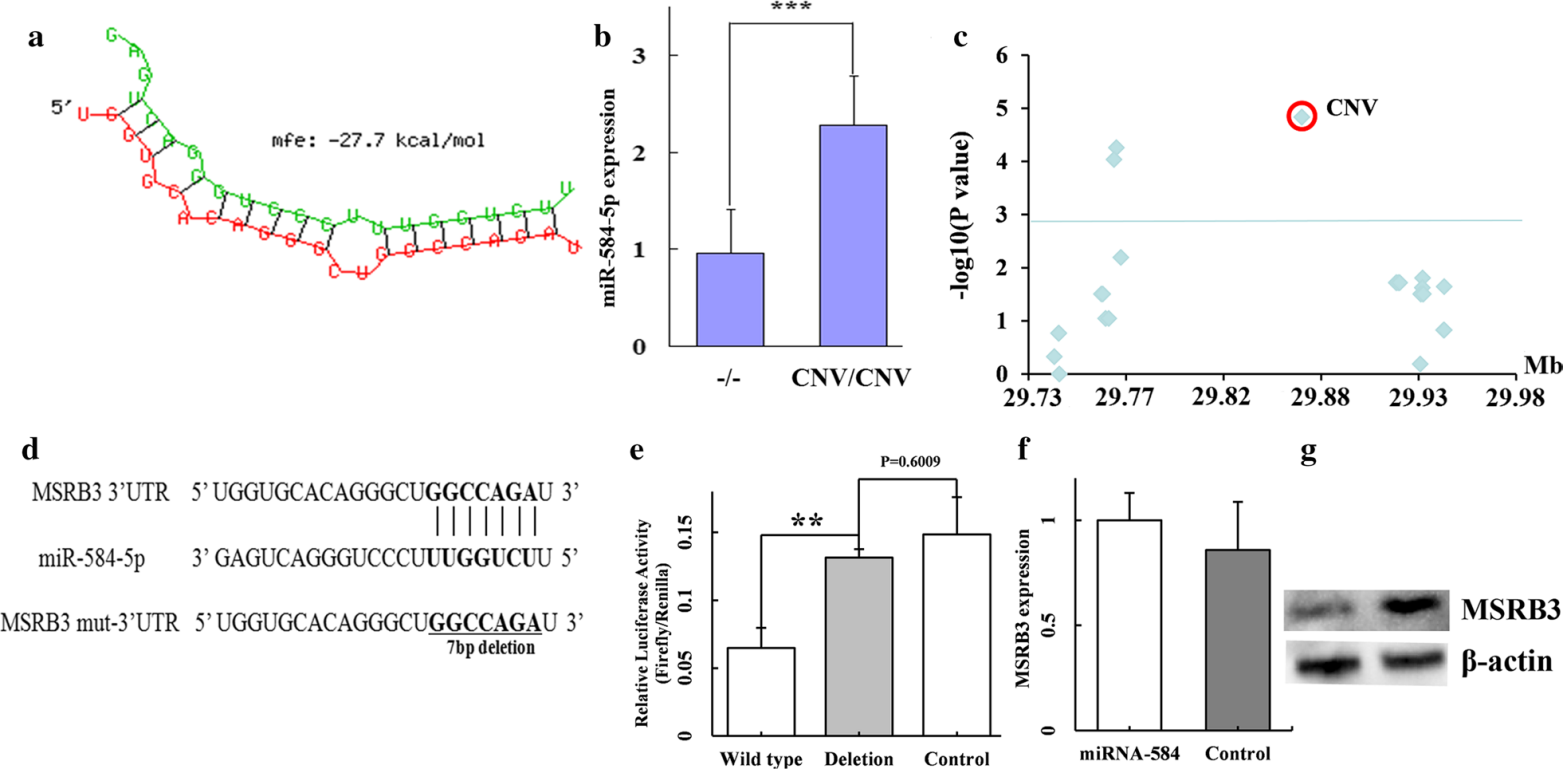
miR-584-5p regulates the expression of *MSRB3* in pigs. **a** A target site of miR-584-5p was identified in the 979–1000 bp region downstream of the stop codon of the *MSRB3* gene via DIANA-microT web server (v5.0). **b** Comparison of the expression levels of miR-584-5p between CNV/CNV and wild-type animals. **c** eQTL mapping for miR-584-5p in ear tissue samples. The CNV shows the strongest association with miR-584-5p expression. **d** Construction of the mutant 3′UTR reporter. The 7-bp seed region (in bold) was deleted in the mutant 3′UTR reporter. **e** Porcine fetal fibroblast cells were transfected with the 3′UTR mutant or wild-type luciferase reporters and co-transfected with miR-584-5p mimics or the negative control duplexes. The relative luciferase activity was measured 48 h later. The figure shows the mean ± S.D. from three independent experiments performed in duplicate (**, *P* < 0.01). The *MSRB3* mRNA expression levels (**f**) and protein levels (**g**) in porcine fetal fibroblast cells transfected with miR-584-5p mimics or negative control duplexes

To establish a relationship between the presence of the CNV and the level of MSRB3, we investigated the genomic region around the 137.85-kb QTL interval and found a novel miRNA (ENSSSCG00000024846, SSC5: 29,938,800–29,938,890 bp), miR-584-5p, which is located on the reverse strand of the reference genome sequence and at 75.21 kb of the 3′ end of *MSRB3*. The CNV is located in the 3′ region of miR-584-5p (Fig. 3d). One target site of this miRNA was predicted to be located within the 3′UTR of *MSRB3*, 979–1000 bp downstream of the stop codon of this gene (Fig. 7a). By comparing the expression levels of miR-584-5p in ear tissue samples between CNV/CNV and wild-type piglets by qRT-PCR, we found that it was significantly higher in CNV/CNV compared to wild-type animals (*P* = 7.82 × 10^−4^, Fig. 7b). eQTL mapping of miR-584-5p in the ear tissue of 59 Landrace piglets showed segregation at the CNV site and confirmed that the CNV had an effect on the expression of miR-584-5p (see Additional file 15: Table S7). We genotyped another 35 SNPs within the 200-kb interval covering the 137.85-kb critical QTL region and the region from the end of the QTL interval to the end of miR-584-5p (29.74–29.94 Mb). These SNPs were considered as candidate SNPs because of their concordance with QTL genotypes in the 19 F_0_ animals of the F_2_ population in the above 59 piglets. SNPs within the CNV were excluded as candidates (described above) and were not genotyped. Interestingly, the CNV showed the strongest association with the expression level of miR-584-5p (*P* = 3.74 × 10^−5^) (Fig. 7c).

Given that the MSRB3 protein level was lower in CNV/CNV compared to wild-type piglets, we hypothesized that miR-584-5p suppresses the expression of MSRB3. To test this hypothesis, we constructed luciferase reporters that included either the wild-type or the mutant 3′UTR of *MSRB3* (Fig. 7d) and co-transfected them with a miR-584-5p mimic or a negative control into porcine fetal fibroblast cells. We found that miR-584-5p significantly reduced the luciferase activity of the wild-type *MSRB3* reporter compared with the negative control (*P *< 0.01). In contrast, we did not observe reduced activity with the mutant luciferase reporter (Fig. 7e), which confirmed that miR-584-5p targets the 3′UTR of *MSRB3* directly. We examined the repression of miR-584-5p in the endogenous MSRB3 protein by transfecting miR-584-5p mimics into porcine fetal fibroblast cells. The results of qRT-PCR indicated that the amount of *MSRB3* mRNA was not decreased by miR-584-5p compared to the control (Fig. 7f). Western blot analysis revealed that miR-584-5p significantly decreased the MSRB3 protein level (*P* < 0.01) (Fig. 7g) (see Additional file 14: Figure S8).

### Other genes that may affect porcine ear size by combining the predicted target genes of miR-584-5p with the differentially expressed genes

Because miR-584-5p can affect the expression of multiple genes, other than *MSRB3*, and because other target genes may be involved in porcine ear size, we predicted the target genes of miR-584-5p in silico with the DIANA-microT web server (v5.0) [39]. At a significance threshold for the miTG score ≥ 0.8, we identified 388 predicted target genes (see Additional file 16: Table S8). To identify other possible target genes associated with porcine ear size, we examined the literature for phenotypic consequences in knockout mice for each of these 388 predicted target genes [42]. As a result, we identified five genes associated with ear morphology, including *FREM2* which is related with abnormal ear shape [42], *LRP6* with abnormal outer ear morphology [42], *FBXO11* [43] and *SIX1* [44] with abnormal middle ear morphology, and *FGF10* with abnormal inner ear morphology [45].

It is well known that miRNA can regulate gene expression by inhibiting target mRNA translation at some stage or by promoting mRNA degradation [46]. To examine whether some of the target genes identified for miR-584-5p were differentially expressed genes (DEG) in the ear tissue between CNV/CNV and wild-type piglets, we isolated a list of 280 DEG with a > 2-fold change at an FDR ≤ 0.001 using RNA sequencing data. These DEG included 82 up-regulated genes and 198 down-regulated genes in the large-eared (CNV/CNV) pigs (see Additional file 17: Table S9). We also examined the literature for phenotypic consequences in knockout mice for these 280 DEG and found abnormal ear morphology in knockout mice for the *COL9A2*, *BMP5*, *ILDR1*, *TYRP1* and *CTGF* genes [47–49]. However, none of these five DEG were target genes of miR-584-5p, and none of the five miR-584-5p target genes described above were DEG. The DEG identified in this study should be located within a transcriptional network involving the miR-584-5p target genes, e.g. *MSRB3*.

## Discussion

In this study, we have narrowed down the interval containing the QTL affecting ear size on SSC5 to 137.85 kb and identified a 38.7-kb CNV as the causative mutation underlying the QTL. We also suggest that this CNV influences porcine ear size by increasing expression of miR-584-5p, which suppresses the expression of its target genes, e.g. *MSRB3*. Considering the universality of this QTL for ear type in pig, sheep and dogs, our findings provide important clues for identifying the causative gene or mutations for ear type in mammals, as well as insights about the development of human external ears as a biomedical model.

Previous studies proposed *LEMD3*, *WIF1,* and *HMGA2* as positional candidate genes for the QTL on SSC5 [5, 7, 10]. Here, fine-mapping analysis allowed us to exclude these three genes as the responsible gene. The use of multiple pig populations allowed us to fine-map the QTL to a small critical interval. In particular, haplotype-sharing analysis in Chinese indigenous pigs with unusually large and floppy ears refined the QTL to a 137.85 kb region. It was previously reported that Chinese indigenous pig breeds show a low level of linkage disequilibrium [50, 51], which facilitates QTL fine-mapping and identification of causative genes for porcine complex traits. In this paper, we provide several solid lines of evidence that support the CNV on SSC5 as a causative mutation: (1) association of this CNV with ear size was strongest in Sutai, Laiwu, DLY and Landrace pigs, which are expected to have a very low level of linkage disequilibrium; (2) the CNV genotypes were completely concordant with the inferred QTL genotypes of all F_0_ individuals in the F_2_ population; and (3) this CNV occurred at very high frequencies in Chinese indigenous pig breeds that have large and floppy ears, but was absent in breeds with small and erect-ears. No other mutations within the 137.85-kb region of the QTL on SSC5 showed such concordance patterns in the tested breeds.

CNV are increasingly shown to have causative effects on phenotypes in livestock [52–56]. Here, we provide another example that a CNV has a major QTL effect on porcine ear size. CNV can account for variation in gene expression in a variety of ways, including through a gene dosage effect, disruption of gene coding regions, and deletion or duplication of regulatory elements [57]. In this study, the CNV is located in the 3′ region of miR-584-5p and was shown to be associated with the expression of miR-584-5p expression by eQTL mapping. We propose two possibilities to explain the mechanism underlying the effect of this CNV on ear size: (1) the presence of this CNV could duplicate the regulatory elements (e.g. enhancer) that affect the expression of miR-584-5p but analysis of the CNV sequence did not identify any potential regulatory elements; and (2) because this CNV is quite large, it could modify the chromatin structure in this region and thereby cause a change in the expression pattern of miR-584-5p.

To investigate how the increased expression of miR-584-5p can lead to enlarged ear size in pigs, we predicted the target genes of miR-584-5p in silico. In addition to *MSRB3*, five of the 388 predicted target genes were associated with abnormal ear morphology in knockout mice. *MSRB3* is within the 137.85-kb QTL interval and functional null mutations of *MSRB3* have been associated with deafness in both humans [58] and mouse [59]. Moreover, knockout of *MSRB3* in mouse inhibits cell growth through the activation of the p53-p21 and p27 pathways [60]. As a proof of principle, we performed experiments in vivo and in vitro to establish that miR-584-5p suppresses the translation of *MSRB3*, which subsequently results in enlarged porcine ear size (both immunohistochemistry and western blot analyses showed that expression of *MSRB3* is associated with ear size). We did not observe a significant difference in *MSRB3* mRNA level between CNV/CNV and wild-type animals. A similar mechanism was also observed in Texel sheep, where a G to A transition in the 3′UTR of the *myostatin* gene creates an illegitimate microRNA target that causes translational inhibition of the gene, which contributes to muscular hypertrophy [61]. Our findings contribute to a better understanding of how the presence of CNV can regulate gene expression. The effects of other target genes of miR-584-5p on porcine ear size requires further investigation. RNA sequencing analysis identified several DEG related to ear morphology in knockout mice but none of these was a target gene of miR-584-5p. These DEG may be located within a transcriptional network involving the miR-584-5p target genes (e.g. *MSRB3*).

## Conclusions

We show that the 38.7-kb CNV in the 3′ region of the *MSRB3* gene is the suggestive causative mutation for a QTL for ear size on SSC5. The presence of this CNV results in an increase in the expression of miR-584-5p, which inhibits the expression of its target gene *MSRB3*, which affects porcine ear size. As a proof of principle, we confirmed that miR-584-5p hinders the translation of *MSRB3* and we found that the MSRB3 protein level differed between pigs with large and small ears. These findings not only shed insight into the underlying mechanisms for development of external ears in mammals but also improve our understanding of the regulation mechanism involving CNV, miRNA, and functional genes.

## Additional files

**Additional file 1: Figure S1.** Ear size and type in different Chinese and Western pig breeds. Pig breeds with large ears: A. Erhualian, B. Laiwu, C. Min, and D. Landrace; Pig breeds with small ears: E. Large White, F. Duroc, G. Tibetan, and H. Wuzhishan.

**Additional file 2: Table S1.** The sample size and final set of informative SNPs for each population.

**Additional file 3: Table S2.** Primers for CNV genotyping.

**Additional file 4: Table S3.** Primers for gene expression analysis.

**Additional file 5: Table S4.** Primers for quantifying the expression profiles of miR-584-5p.

**Additional file 6: Figure S2.** Quantile–quantile plot of SNPs after quality control in genome-wide association studies for porcine ear size.

**Additional file 7: Figure S3.** Correction of the assembly error in the pig reference genome sequence within the critical QTL region by blast analysis with the human orthologous region.

**Additional file 8: Figure S4.** Transcript isoforms of the MSRB3 gene identified in this study. The figure indicates the position of each exon in the porcine genome assembly 11.1. The mRNA sequences of the two transcript isoforms were submitted to NCBI with accession numbers KX557289 and KX557290.

**Additional file 9: Table S5.** Allele frequencies of the mutations for which the genotypes in F_0_ animals were in concordance with the QTL genotypes in large- and small-eared pigs.

**Additional file 10: Table S6.** Copy numbers of the CNV in different Chinese indigenous pig breeds with large and floppy ears estimated by droplet digital PCR and qPCR.

**Additional file 11: Figure S5.** Conditional association study between the SNPs on SSC5 and porcine ear size by treating the genotypes of the CNV as fixed effects. The *Y*-axis shows –log 10 (P) values obtained in association studies and the *X*-axis indicates the locations of the CNV and SNPs; (a), (b) and (c) indicate the Manhattan plots of association analyses in which the genotypes of the CNV in Laiwu, DLY and Sutai population were treated as fixed effects.

**Additional file 12: Figure S6.** Expression profiles of *HMGA2*, *LEMD3* and the transcript variants of MSRB3 in ear tissues between CNV/CNV and wild-type piglets. *HMGA2*, *LEMD3* and the two transcript isoforms of *MSRB3* showed no significant difference in expression levels between CNV/CNV and wild-type piglets.

**Additional file 13: Figure S7.** Gene structure analysis of *MSRB3* with RNA sequencing data. RNA sequencing analysis did not identify any distinct gene structures (alternative splicing and gene boundary) associated with ear size. The figure only shows the last two exons of *MSRB3*, which are covered by the CNV.

**Additional file 14: Figure S8.** The expression level of *MSRB3* across tissue and cell types. (a) The expression level of *MSRB3* in six different tissue types by RT-PCR. (b) Western blot analysis showing MSRB3 protein levels in ear tissues of CNV/CNV, CNV/− and −/− pigs. (c) The MSRB3 protein levels in porcine fetal fibroblast cells transfected with miR-584-5p mimics or negative control duplexes.

**Additional file 15: Table S7.** CNV distribution in 59 Landrace pigs used for eQTL mapping of miR-584-5p.

**Additional file 16: Table S8.** Predicted target genes of miR-584-5p in silico.

**Additional file 17: Table S9.** Differentially expressed genes identified in ear tissue between CNV/CNV and wild-type animals.
