# Supplementary figures and images for: Copy number variation in the MSRB3 gene enlarges porcine ear size through a mechanism involving miR-584-5p
Source: Genet Sel Evol. 2018 Dec 27;50:72. doi: 10.1186/s12711-018-0442-6 (PMC6307293; doi:10.1186/s12711-018-0442-6)

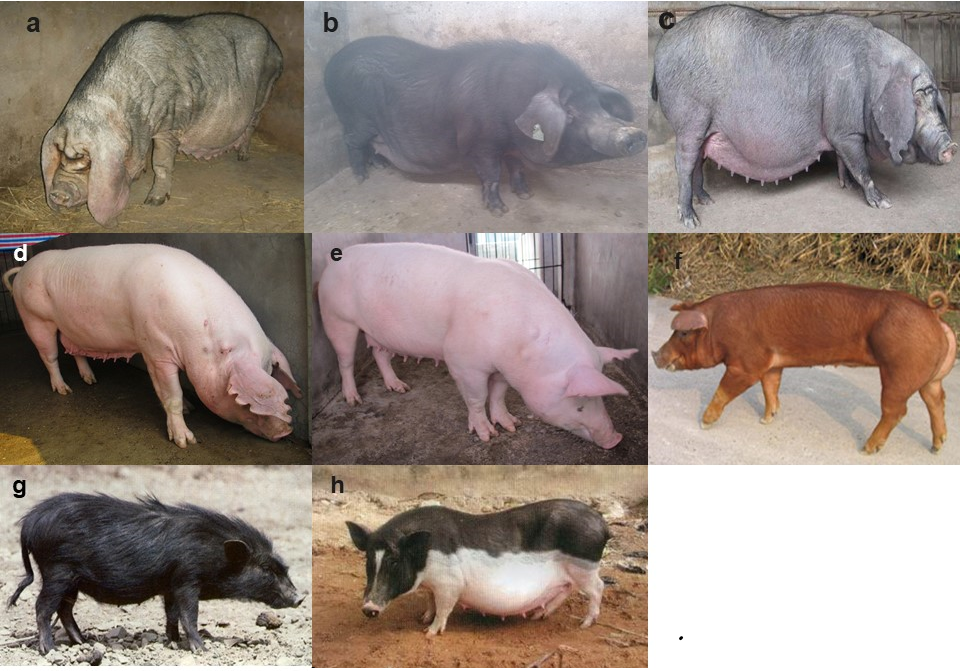

Supplement: Supplementary file 1 — Additional file 1: Figure S1. Ear size and type in different Chinese and Western pig breeds. Pig breeds with large ears: A. Erhualian, B. Laiwu, C. Min, and D. Landrace; Pig breeds with small ears: E. Large White, F. Duroc, G. Tibetan, and H. Wuzhishan. [file 12711_2018_442_MOESM1_ESM.tif]

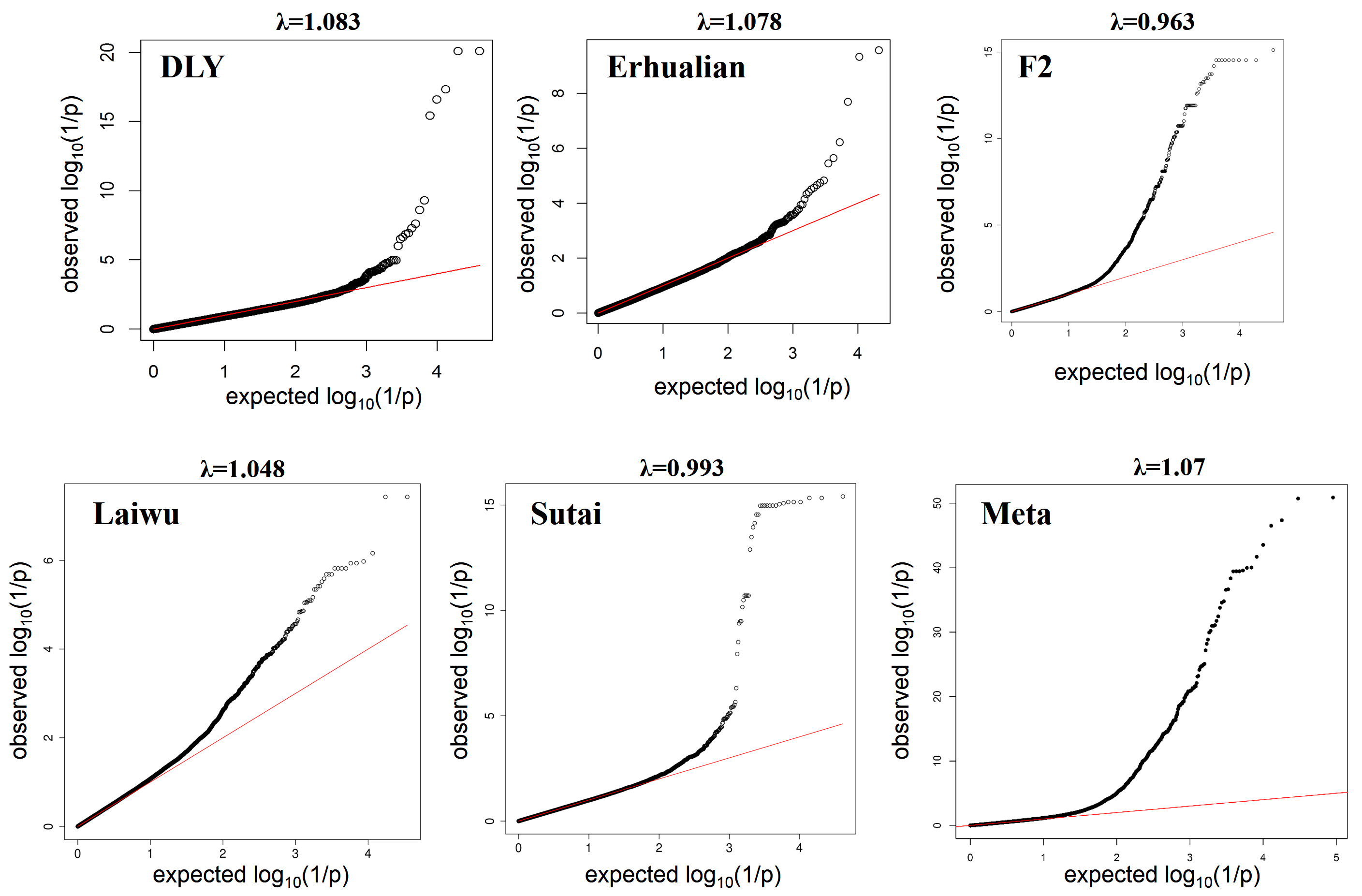

Supplement: Supplementary file 6 — Additional file 6: Figure S2. Quantile–quantile plot of SNPs after quality control in genome-wide association studies for porcine ear size. [file 12711_2018_442_MOESM6_ESM.tif]

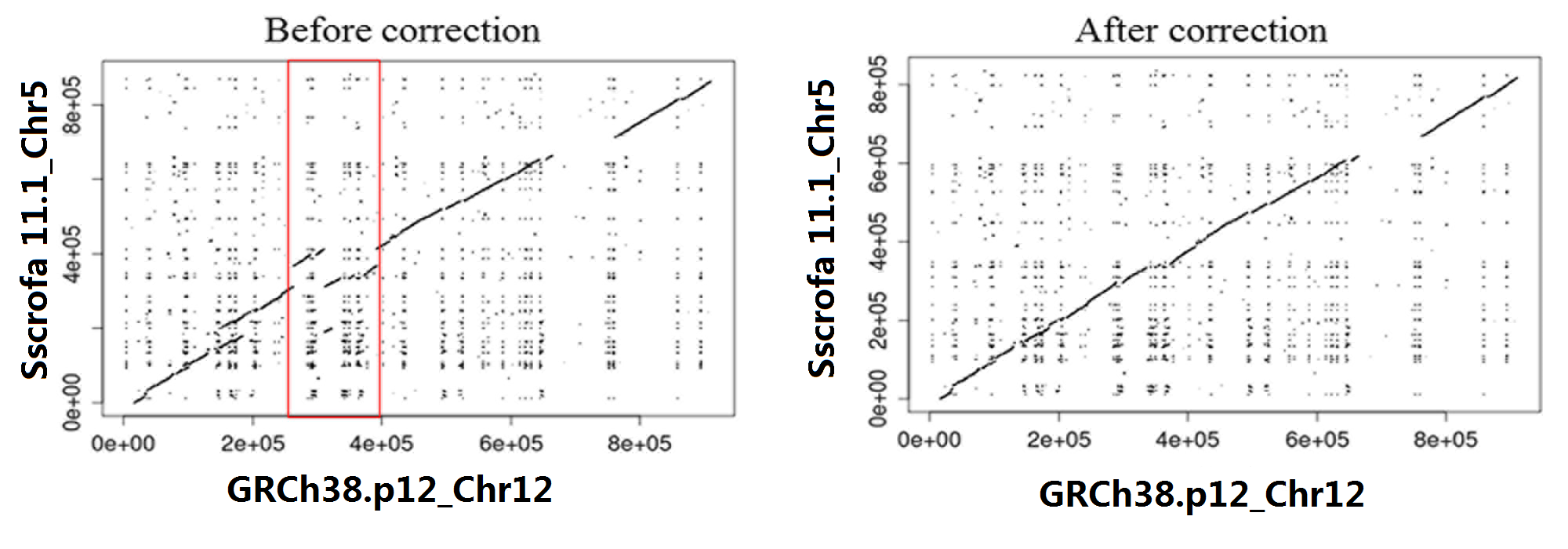

Supplement: Supplementary file 7 — Additional file 7: Figure S3. Correction of the assembly error in the pig reference genome sequence within the critical QTL region by blast analysis with the human orthologous region. [file 12711_2018_442_MOESM7_ESM.tif]

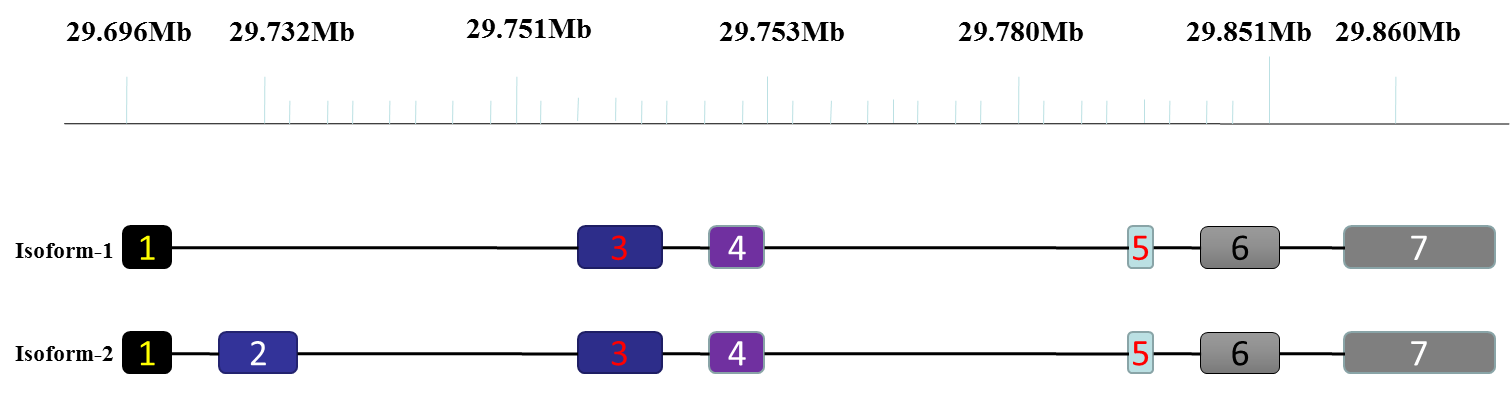

Supplement: Supplementary file 8 — Additional file 8: Figure S4. Transcript isoforms of the MSRB3 gene identified in this study. The figure indicates the position of each exon in the porcine genome assembly 11.1. The mRNA sequences of the two transcript isoforms were submitted to NCBI with accession numbers KX557289 and KX557290. [file 12711_2018_442_MOESM8_ESM.tif]

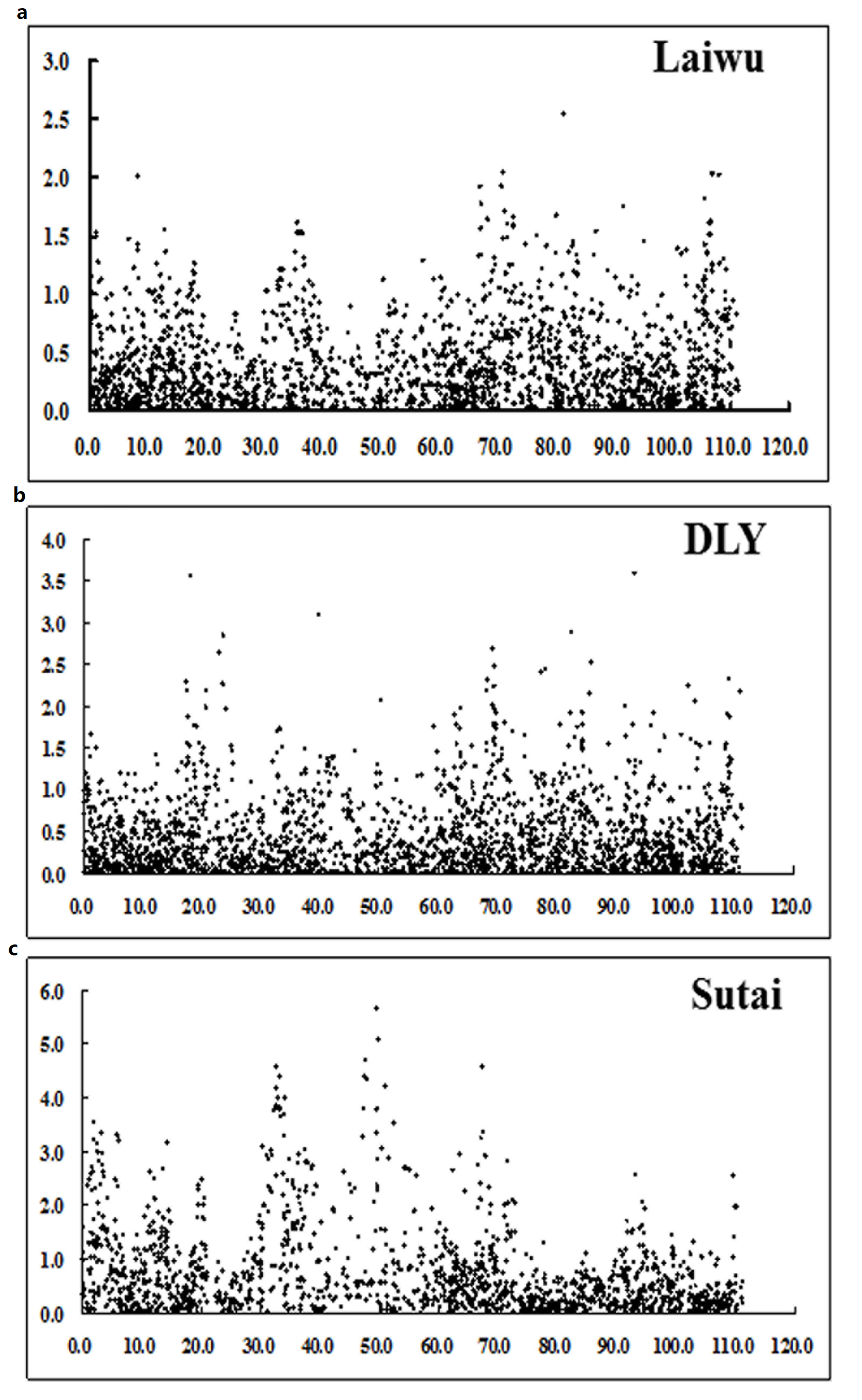

Supplement: Supplementary file 11 — Additional file 11: Figure S5. Conditional association study between the SNPs on SSC5 and porcine ear size by treating the genotypes of the CNV as fixed effects. The Y-axis shows –log 10 (P) values obtained in association studies and the X-axis indicates the locations of the CNV and SNPs; (a), (b) and (c) indicate the Manhattan plots of association analyses in which the genotypes of the CNV in Laiwu, DLY and Sutai population were treated as fixed effects. [file 12711_2018_442_MOESM11_ESM.tif]

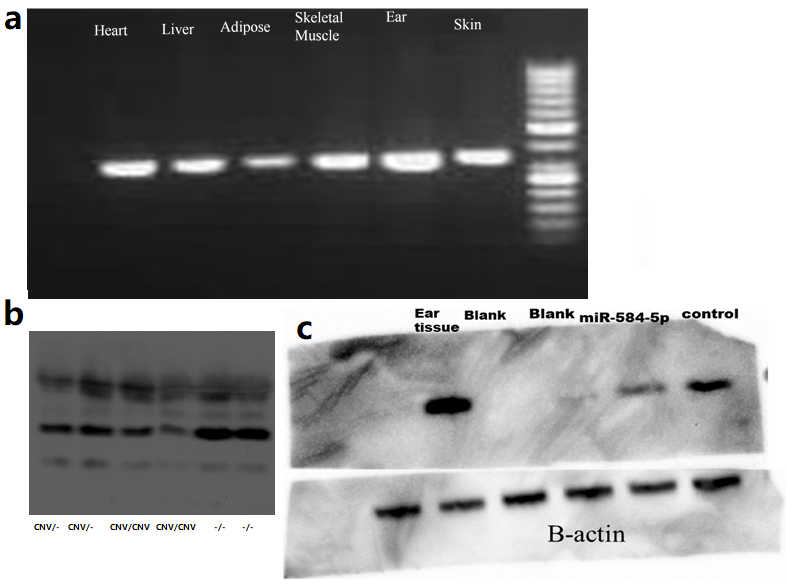

Supplement: Supplementary file 14 — Additional file 14: Figure S8. The expression level of MSRB3 across tissue and cell types. (a) The expression level of MSRB3 in six different tissue types by RT-PCR. (b) Western blot analysis showing MSRB3 protein levels in ear tissues of CNV/CNV, CNV/− and −/− pigs. (c) The MSRB3 protein levels in porcine fetal fibroblast cells transfected with miR-584-5p mimics or negative control duplexes. [file 12711_2018_442_MOESM14_ESM.tif]
