# Supplementary material for: Copy number variation in the MSRB3 gene enlarges porcine ear size through a mechanism involving miR-584-5p
Source: Genet Sel Evol. 2018 Dec 27;50:72. doi: 10.1186/s12711-018-0442-6 (PMC6307293; doi:10.1186/s12711-018-0442-6)
Supplement: Supplementary file 2 — Additional file 2: Table S1. The sample size and final set of informative SNPs for each population. [file 12711_2018_442_MOESM2_ESM.doc]

**Table S1. The sample size and final set of informative SNPs for each population**

| **Population** | **Sample size** | **Informative SNPs** | **Genome-wide significant threshold** |
| --- | --- | --- | --- |
| White Duroc × Erhualian F_2_ | 912 | 44,122 | 1.13 × 10^-6^ |
| Sutai | 403 | 44,875 | 1.11 × 10^-6^ |
| Laiwu | 312 | 43,236 | 1.16 × 10^-6^ |
| DLY | 343 | 51,642 | 9.68 × 10^-7^ |
| Erhualian | 331 | 3,3094 | 1.51 × 10^-6^ |
