# Supplementary material for: Copy number variation in the MSRB3 gene enlarges porcine ear size through a mechanism involving miR-584-5p
Source: Genet Sel Evol. 2018 Dec 27;50:72. doi: 10.1186/s12711-018-0442-6 (PMC6307293; doi:10.1186/s12711-018-0442-6)
Supplement: Supplementary file 5 — Additional file 5: Table S4. Primers for quantifying the expression profiles of miR-584-5p. [file 12711_2018_442_MOESM5_ESM.doc]

| **Primer Name** | **Primer sequence** | **Amplified Fragment (bp)** | **Annealing Temperature (^0^C)** |
| --- | --- | --- | --- |
| miR-584-5p Primer FP | 5’-AACACGCTTCTGGTTTCCCT-3’ | 73 | 60 |
| miR-584-5p Primer RP | 5’-GTCGTATCCAGTGCAGGGT-3’ |
| miR-584-5p-Probe | FAM-5’-ACTGGATACGACCTCAGT-3’- MGBNFQ |
| U6 Primer FP | 5’-GCTTCGGCAGCACATATACTAA-3’ | 82 | 60 |
| U6 Primer RP | 5’-CGAATTTGCGTGTCATCCTT-3’ |
| U6 Probe | FAM-5’-GCTCAGGGGCCATGCTAATCTTCTC-3’ -MGBNFQ |
| Primer for reverse transcription of U6 | 5’-CGAATTTGCGTGTCATCCTT-3’ | - | - |
| Primer for reverse transcription of miR-584 | 5’-GTCGTATCCAGTGCAGGGTCCGAGGTATTCGCACT  GGATACGACCTCAGT-3’, | - | - |

**Table S4. Primers for quantifying the expression profiles of miR-584-5p**
