# Supplementary material for: Copy number variation in the MSRB3 gene enlarges porcine ear size through a mechanism involving miR-584-5p
Source: Genet Sel Evol. 2018 Dec 27;50:72. doi: 10.1186/s12711-018-0442-6 (PMC6307293; doi:10.1186/s12711-018-0442-6)
Supplement: Supplementary file 9 — Additional file 9: Table S5. Allele frequencies of the mutations for which the genotypes in F0 animals were in concordance with the QTL genotypes in large- and small-eared pigs. [file 12711_2018_442_MOESM9_ESM.doc]

Table S5. Allele frequencies of the mutations for which genotypes in F_0_ animals were concordance with QTL genotypes in large- and small-eared pigs

| **Polymorphism position** | **allele increasing ear size** | **Small-eared pigs** | |  | **Large-eared pigs** | |
| --- | --- | --- | --- | --- | --- | --- |
|  |  | **Wuzhishan (n = 6)** | **Tibetan (n = 24)** |  | **Min**  **(n = 6)** | **Erhualian**  **(n = 14)** |
| 29738849 | A | 0.92 | 0.92 |  | 1 | 1 |
| 29740916 | A | 0.92 | 0.81 |  | 1 | 1 |
| 29741061 | A | 0.92 | 0.81 |  | 1 | 1 |
| 29741063 | T | 0.92 | 0.81 |  | 1 | 1 |
| 29741065 | C | 0.92 | 0.81 |  | 1 | 1 |
| 29741338 | indel | 0.42 | 0.65 |  | 1 | 1 |
| 29808405 | A | 0.33 | 0.17 |  | 1 | 1 |
| 29809064 | C | 0.33 | 0.19 |  | 1 | 1 |
| 29810621 | A | 0.33 | 0.17 |  | 1 | 1 |
| 29812180 | G | 0.17 | 0.15 |  | 1 | 1 |
| 29814883 | T | 0.33 | 0.17 |  | 1 | 1 |
| 29816100 | C | 0.50 | 0.23 |  | 1 | 1 |
| 29818367 | A | 0.33 | 0.17 |  | 1 | 1 |
| 29831326 | C | 0.42 | 0.17 |  | 1 | 1 |
| 29832963 | G | 0.17 | 0.17 |  | 1 | 1 |
| 29839916 | indel | 0.33 | 0.19 |  | 1 | 1 |
| 29840579 | A | 0.33 | 0.17 |  | 1 | 1 |
| 29841538 | T | 0.33 | 0.17 |  | 1 | 1 |
| 29745362 | T | 0.75 | 0.75 |  | 1 | 1 |
| 29745783 | A | 0.33 | 0.65 |  | 1 | 1 |
| 29748991 | indel | 0.33 | 0.65 |  | 1 | 1 |
| 29749352 | C | 0.33 | 0.65 |  | 1 | 1 |
| 29750365 | T | 0.33 | 0.65 |  | 1 | 1 |
| 29750638 | C | 0.58 | 0.69 |  | 1 | 1 |
| 29750681 | A | 0.58 | 0.75 |  | 1 | 1 |
| 29751013 | T | 0.58 | 0.75 |  | 1 | 1 |
| 29752112 | T | 0.33 | 0.65 |  | 1 | 1 |
| 29752627 | C | 0.58 | 0.75 |  | 1 | 1 |
| 29752653 | indel | 0.33 | 0.58 |  | 1 | 1 |
| 29753139 | T | 0.58 | 0.75 |  | 1 | 1 |
| 29753703 | T | 0.33 | 0.60 |  | 1 | 1 |
| 29754851 | C | 0.50 | 0.75 |  | 1 | 1 |
| 29754938 | indel | 0.58 | 0.75 |  | 1 | 1 |
| 29755223 | G | 0.58 | 0.75 |  | 1 | 1 |
| 29755384 | T | 0.33 | 0.67 |  | 1 | 1 |
| 29755680 | T | 0.42 | 0.75 |  | 1 | 1 |
| 29756036 | C | 0.42 | 0.75 |  | 1 | 1 |
| 29756129 | A | 0.33 | 0.60 |  | 1 | 1 |
| 29756157 | G | 0.42 | 0.75 |  | 1 | 1 |
| 29756167 | A | 0.42 | 0.75 |  | 1 | 1 |
| 29756176 | C | 0.42 | 0.75 |  | 1 | 1 |
| 29756202 | A | 0.42 | 0.75 |  | 1 | 1 |
| 29756270 | C | 0.42 | 0.75 |  | 1 | 1 |
| 29756292 | T | 0.42 | 0.75 |  | 1 | 1 |
| 29756591 | A | 0.42 | 0.75 |  | 1 | 1 |
| 29756614 | A | 0.42 | 0.75 |  | 1 | 1 |
| 29756676 | G | 0.33 | 0.60 |  | 1 | 1 |
| 29756789 | G | 0.33 | 0.60 |  | 1 | 1 |
| 29756892 | A | 0.42 | 0.75 |  | 1 | 1 |
| 29756973 | G | 0.42 | 0.75 |  | 1 | 1 |
| 29757028 | A | 0.42 | 0.75 |  | 1 | 1 |
| 29757118 | A | 0.33 | 0.60 |  | 1 | 1 |
| 29757185 | T | 0.42 | 0.75 |  | 1 | 1 |
| 29757187 | T | 0.42 | 0.75 |  | 1 | 1 |
| 29759043 | C | 0.42 | 0.75 |  | 1 | 1 |
| 29759044 | A | 0.33 | 0.60 |  | 1 | 1 |
| 29759144 | A | 0.42 | 0.75 |  | 1 | 1 |
| 29761841 | indel | 0.33 | 0.67 |  | 1 | 1 |
| 29762936 | T | 0.33 | 0.67 |  | 1 | 1 |
| 29763960 | T | 0.33 | 0.60 |  | 1 | 1 |
| 29770148 | C | 0.50 | 0.29 |  | 1 | 1 |
| 29848739 | C | 0.33 | 0.17 |  | 1 | 1 |
| 29858427 | A | 0.33 | 0.19 |  | 1 | 1 |
| 29858812 | T | 0.17 | 0.15 |  | 1 | 1 |
| 29695369* | T | 91.67 | 87.50 |  | 1 | 1 |
| 29862412* | C | 91.67 | 95.83 |  | 1 | 1 |
| CNV | duplication | 0 | 0 |  | 1 | 1 |

Note: * The SNPs reported by Zhang *et al*. (2015)
