# Supplementary material for: Copy number variation in the MSRB3 gene enlarges porcine ear size through a mechanism involving miR-584-5p
Source: Genet Sel Evol. 2018 Dec 27;50:72. doi: 10.1186/s12711-018-0442-6 (PMC6307293; doi:10.1186/s12711-018-0442-6)
Supplement: Supplementary file 10 — Additional file 10: Table S6. Copy numbers of the CNV in different Chinese indigenous pig breeds with large and floppy ears estimated by droplet digital PCR and qPCR. [file 12711_2018_442_MOESM10_ESM.doc]

Table S6. Copy numbers of the CNV in different Chinese indigenous pig breeds with large and floppy ears by droplet digital PCR and qPCR

| **Sample** | **ddPCR** | **RQ** |
| --- | --- | --- |
| Laiwu1013 | 6 | 3.03 |
| Laiwu1076 | 6 | 3.27 |
| Erhualian90 | 4 | 1.83 |
| Erhualian74 | 4 | 2.43 |
| MIN307 | 6 | 3.18 |
| MIN312 | 6 | 3.15 |
| Mi1 | 4 | 1.86 |
| Mi2 | 4 | 1.79 |
| Meishan1 | 4 | 1.97 |
| Meishan2 | 4 | 1.74 |
| Jiaxing Black1 | 4 | 2.02 |
| Jiaxing Black2 | 4 | 2.50 |
